# Alternatively spliced exon regulates context-dependent MEF2D higher-order assembly during myogenesis

**DOI:** 10.1038/s41467-023-37017-7

**Published:** 2023-03-10

**Authors:** Mónika Gönczi, João M. C. Teixeira, Susana Barrera-Vilarmau, Laura Mediani, Francesco Antoniani, Tamás Milán Nagy, Krisztina Fehér, Zsolt Ráduly, Viktor Ambrus, József Tőzsér, Endre Barta, Katalin E. Kövér, László Csernoch, Serena Carra, Monika Fuxreiter

**Affiliations:** 1 Department of Physiology, Faculty of Medicine, University of Debrecen, Egyetem tér 1, H-4032 Debrecen, Hungary; 2 ELKH Cell Physiology Research Group, Department of Physiology, Faculty of Medicine, University of Debrecen, Egyetem tér 1, H-4032 Debrecen, Hungary; 3 Department of Biomedical Sciences, University of Padova, Via Ugo Bassi 58/B, 35131 Padova, Italy; 4 Department of Biomedical, Metabolic and Neural Sciences, University of Modena and Reggio Emilia G. Campi 287, 41125 Modena, Italy; 5 Department of Inorganic and Analytical Chemistry, University of Debrecen, Egyetem tér 1, H-4032 Debrecen, Hungary; 6 MTA-DE Molecular Recognition and Interaction Research Group, University of Debrecen Egyetem tér 1, H-4032 Debrecen, Hungary; 7 Department of Biochemistry and Molecular Biology, Faculty of Medicine, University of Debrecen, Egyetem tér 1, H-4032 Debrecen, Hungary; 8 Department of Genetics and Genomics, Institute of Genetics and Biotechnology, Hungarian University of Agriculture and Life Sciences, Szent-Györgyi A. út 4, H-2100 Gödöllő, Hungary

**Keywords:** Computational biology and bioinformatics, Cell biology, Structural biology

## Abstract

During muscle cell differentiation, the alternatively spliced, acidic β-domain potentiates transcription of Myocyte-specific Enhancer Factor 2 (Mef2D). Sequence analysis by the FuzDrop method indicates that the β-domain can serve as an interaction element for Mef2D higher-order assembly. In accord, we observed Mef2D mobile nuclear condensates in C2C12 cells, similar to those formed through liquid-liquid phase separation. In addition, we found Mef2D solid-like aggregates in the cytosol, the presence of which correlated with higher transcriptional activity. In parallel, we observed a progress in the early phase of myotube development, and higher MyoD and desmin expression. In accord with our predictions, the formation of aggregates was promoted by rigid β-domain variants, as well as by a disordered β-domain variant, capable of switching between liquid-like and solid-like higher-order states. Along these lines, NMR and molecular dynamics simulations corroborated that the β-domain can sample both ordered and disordered interactions leading to compact and extended conformations. These results suggest that β-domain fine-tunes Mef2D higher-order assembly to the cellular context, which provides a platform for myogenic regulatory factors and the transcriptional apparatus during the developmental process.

## Abstract (web summary)

Mef2D context-dependent higher-order assembly controls simultaneous interactions with the basal machinery and the myogenic factors to activate distinct gene expression programs for muscle lineage development.

## Introduction

Myogenic lineage establishment and maintenance of terminal myogenic phenotype is controlled by precise dynamics of temporal and spatial expression, and hierarchical relationship between four myogenic regulatory factors (MRFs)^1^. Myocyte-specific Enhancer Factor 2 (Mef2) protein belongs to the MADS (MCM1-Agamous-Deficiens-serum response factor) box family of transcription factors, which potentiate the transcriptional activity of myogenic proteins^2^. Mef2 activity is precisely controlled during muscle development; in addition, the four isoforms encoded by separated genes in vertebrates further increase its functional variety^3^. The four *MEF2* genes (*MEF2A*, *-B*, *-C* and *-D*) have spatially and temporally distinct expression during development^4^. Loss-of-function studies showed that mice lacking *MEF2A* and *MEF2C* were not viable owing to cardiac dysfunctions, however, *MEF2D* null mice were viable with cardiac defects^5^.

Mef2 is activated by myogenic regulatory factors^6,7^ and the synergy with basic helix-loop-helix (bHLH) proteins is critical for regulating the expression of muscle-specific genes^8^. The interactions between Mef2 and myogenic proteins appears to be rather complex, involving a dimerisation of Mef2 as well as its bHLH/E12 partners^9^. A physical interaction between these dimers through the DNA-binding and dimerisation motifs has been proposed^8^. While bound to DNA, the MyoD basic region was shown to mediate the protein-protein interactions with Mef2^10^. Mutations of two basic residues of MyoD bHLH region, for example, impeded interactions with Mef2 and resulted in myogenic inactivity^11^. The proximity of these proteins on DNA and their higher local concentrations contribute to transcriptional synergy during myogenesis^10^.

**Fig. 1:**
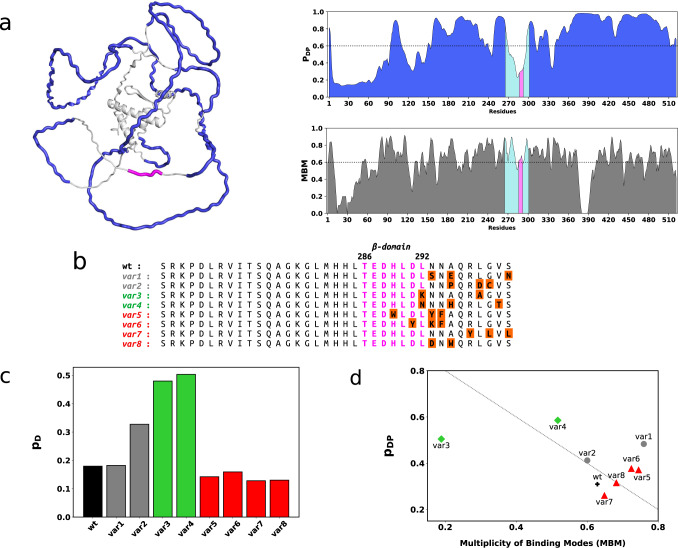
Predicted structure and dynamics characteristics of the wild-type Mef2D and variant sequences. **a** Mef2D is extensively disordered and predicted to form liquid-liquid phase separated condensates. The Mef2D structure predicted by Alphafold^57^ indicates a small structured domain involving the N-terminal ~100 residues and most of the transactivation domain (TAD) contains is disordered. The regions promoting formation of liquid-like droplets by the FuzDrop method^58^ are marked by blue. The β-domain (magenta) appears as an ordered motif within the disordered transactivation region. FuzDrop predictions^26^ shown on the right panels indicate high droplet-promoting probability (p_DP_) in particular for regions 155-268 residues and 341-520 residues, which are predicted to spontaneously form liquid-liquid phase separated condensates. The β-domain (magenta) and its flanking regions (cyan) are predicted to serve as ordered interaction motifs within the condensate (see also Supplementary Fig. 1). In addition, the β-domain region is capable of sampling a multiplicity of binding modes (MBM), indicating its sensitivity to the cellular context. **b** Sequences of the designed Mef2D variants. The β-domain and its flanking regions are shown for the wild-type (**wt**) Mef2D (UniProt code: Q14814; https://legacy.uniprot.org/uniprot/Q14814; 265-301 residues), **var1** and **var2** with similar β-domain dynamics (gray), **var3** and **var4** with mobile β-domains (green), **var5**-**var8** with rigid β-domains (red) as compared to wild-type Mef2D. The sequence of the β-domain is magenta, mutated residues (orange) are highlighted. **c** Predicted β-domain disorder of Mef2D variants. Structural disorder in the unbound state of Mef2D were computed for the full protein sequence using the Espritz method^30^ as embedded in the FuzPred program^25^ and the p_D_ values were averaged for residues 286-292. The **var3** and **var4** variants (green) are above the threshold between disorder and order (p_D_ ≥ 0.3085^30^). **var1** (gray) has similar, **var2** (gray) has slightly more mobile β-domain than the wild-type Mef2D (black). **var5**-**var8** variants (red) are predicted to have more rigid β-domain than the wild-type. **d** Droplet landscape of the Mef2D variants. The droplet landscape shows the droplet probability (p_DP_) as a function of the multiplicity of binding modes (MBM)^31,35^. The assemblies below the diagonal are likely more solid-like, those above the diagonal are more liquid like^33^. High MBM values indicate an increased likelihood to change between liquid-like and solid-like forms, for example in case of **var8**. More mobile β-domain variants (**var3**, **var4**, green diamonds) exhibit increased probability to form droplets (higher p_DP_), whereas more rigid β-domain variants (**var5**-**var8**, red triangles) more likely form solid-like states depending on the cellular conditions (high MBM).

Tissue-specific alternative splicing further regulates the activity of Mef2 proteins during muscle differentiation^3,12^. For example, the inclusion of a short acidic region, termed as the β-domain was in particular observed to potentiate transcription in all *MEF2* gene products as compared to the splicing variants lacking this segment. The β-domain function was position-independent suggesting an autonomous transcriptional activity, not related to DNA binding or dimerisation^13^. Tissue-specific alternative splicing of short, disordered motifs often rewires protein interaction networks^14,15^. In accord, the alternative inclusion of the β-domain was proposed to influence the coordination with myogenic proteins during differentiation. The characteristic feature of this seven-residue segment is the abundance of acidic residues, the mutation of which abolishes transcription^13^. Therefore, the β-domain was proposed to serve as an acidic blob^16^ within the Mef2 disordered transactivation domain (TAD, Fig. 1a), which may directly interact with the basal machinery. These acidic blobs function without strong structure and sequence constraints^17^ through a multitude of alternative interactions formed between the transcription factor and its partner^18^. Growing structural data on transcriptional assemblies^19,20^ support that interaction plasticity is beneficial for fine-tuned regulation of the transcription machinery^21^. In addition, transcription regulation is also enabled by higher-order organisation of proteins^22,23^, including a wide range of non-stoichiometric clusters^24^.

Based on these insights, we reasoned that the β-domain may influence the higher-order organisation of Mef2 proteins, enabling their coordination with both the myogenic regulatory factors and the basal machinery. To investigate how alternative inclusion of the β-domain contributes to myogenic transcription, we designed eight Mef2D variants with distinct β-domain properties using a combination of FuzPred method of predicting disordered interactions^25^ and the FuzDrop method of predicting the probability of forming protein condensates^26^. The variants with similar (***var1***, ***var2***), mobile (***var3***, ***var4***) and rigid (***var5*** – ***var8***) β-domains as compared to the wild-type Mef2D (Figs. 1b, 1c) were also predicted to have different cellular behaviors and higher-order assembly (Fig. 1d). We in particular anticipated that the Mef2D variants with different β-domain dynamics will respond differently to changes in the cellular conditions during myogenesis. Then we have assessed the transcriptional activity of the Mef2D variants in reference to the wild-type protein and the isoform lacking the β-domain, termed as β-. We found a negative relationship between the β-domain dynamics and the transcriptional activity; more rigid variants were more potent to increase transcriptional activity in C2C12 cells. In parallel, we observed that more rigid variants, in particular ***var8*** progressed the early phase of differentiation. Then we have characterised how β-domain dynamics affects Mef2D higher-order assembly. In accord with the predictions, we found that Mef2D formed nuclear puncta in C2C12 cells, the mobility of which resembled liquid-liquid phase separated condensates. In addition, we also observed solid-like aggregates in the cytosol, in particular promoted by a rigid β-domain variant (***var8***). Strikingly, solid aggregates were also formed by the mobile ***var4***, which was predicted to undergo liquid-liquid phase separation as well as to readily convert between liquid-like and solid-like higher-order states (Fig. 1d). NMR and molecular dynamics simulations confirmed that depending on its flanking regions, the β-domain samples both ordered interactions, which can lead to solid-like aggregates as well as disordered interactions, which can result in liquid-like condensates. Based on these results we reason that the alternatively spliced β-domain serves as an interaction element for Mef2D higher-order assembly, which is required for transcription potentiation and differentiation. The characteristics of the assembly are fine-tuned by β-domain dynamics so that Mef2D can efficiently communicate with both the myogenic regulatory proteins and the basal machinery.

## Results

### Designing Mef2D variants with altered β-domain dynamics and higher-order assembly

Proteins sample a wide range of states from ordered to disordered conformations both in free and bound forms^27,28^ and variations of structure and interaction patterns lead to functional plasticity^29^. Most of the transactivation region (TAD) of Mef2D is predicted to be disordered by the Espritz method^30^ in the free form, whereas the β-domain and its flanking regions appear to be more structured (Supplementary Fig. 1a). We used the FuzPred method to estimate the interaction behaviors, as this approach does not require the knowledge of the specific partner^25^. These predictions indicate that the majority of the TAD region forms heterogeneous, disordered interactions^31^, whereas the β-domain serves as a more ordered recognition element (Supplementary Fig. 1b), which can establish well-defined contacts with the partner. In addition, a few motifs, (239–247, 331–339, 355–363, 427–434 residues, Supplementary Fig. 1b) are predicted to form ordered interactions, out of which K245 was identified as an acetylation site^32^. Regions, which form disordered interactions through heterogeneous and variable contacts, often promote liquid-liquid phase separation^26^. Based on FuzDrop method^26^, the majority of the Mef2D TAD is predicted to promote formation of liquid-liquid phase separated condensates (Fig. 1a). The β-domain, however, cannot spontaneously form condensates due to structural order (Fig. 1a). On the other hand, the β-domain is estimated to have a multiplicity of binding modes (MBM, Fig. 1a), which suggests a change between ordered and disordered interactions, the latter facilitating Mef2D partitioning in condensates^33^.

We used three residue-specific dynamics characteristics to design β-domain variants: *i)* probability of structural disorder in the free state (*p*_*D*_, computed by the Espritz method^30^); *ii)* probability of disordered interactions (*p*_*DD*_, computed by the FuzPred method^25^); *iii)* droplet-promoting probability as predicted by the FuzDrop method (*p*_*DP*_, computed by the FuzDrop method^26^). Then we averaged these values for the β-domain (286-292 residues) and assessed the impact of mutations on three dynamics parameters (*p*_*D*_, *p*_*DD*_, *p*_*DP*_) using structural disorder as the primary ranking (Methods). Then we combined mutations to increase the impact on β-domain dynamics and higher-order assembly. Two variants with similar β-domain dynamics to the wild-type (***var1***, ***var2***) had additional negatively charged residues introduced at the C-terminal flanking region (Figs. 1b, 1c). Two variants with mobile β-domains (***var3***, ***var4***) contained positively charged and polar residues (Figs. 1b, 1c). The four variants with rigid β-domains (***var5*** – ***var8***) contained more aromatic and hydrophobic residues (Figs. 1b, 1c).

In addition, we also predicted how the variants affect Mef2D higher-order assembly based on the droplet-promoting probability (*p*_*DP*_), which characterises the ability to spontaneously undergo liquid-liquid phase separation, and the multiplicity of binding modes (MBM), which indicates the likelihood of changing between liquid-like and solid-like states^31^. The complex cellular behavior is represented on the droplet landscape^34,35^, which displays the droplet-probability as a function of the MBM^33^ (Fig. 1d). For example, ***var3*** and ***var4*** both have disordered β-domain (Fig. 1c, Supplementary Table 1), yet they are predicted to exhibit different sensitivity to the cellular context. ***var3*** has a positively charged lysine at position 292, which preferably forms disordered interactions and liquid-like condensates, almost independently on other cellular factors (low MBM, Fig. 1d). In contrast, ***var4*** has an asparagine at position 292, which forms a NNN motif with the neighboring residues. This asparagine motif can sample both disordered and ordered interactions and thus is predicted to form liquid-like condensate or solid-like state depending on the cellular conditions (high MBM, Fig. 1d)^33^. Similarly, introducing an asparagine into the C-terminal flanking region of ***var1*** or adding aromatic groups to the β-domain of ***var5*** and ***var6*** also increases the sensitivity to the cellular conditions (Fig. 1d). Introducing both a negative and aromatic residue in ***var8*** results in comparable cellular sensitivity to the wild-type protein (Fig. 1d). Interestingly, ***var8*** is located at the diagonal of the droplet landscape, suggesting that the transition from droplet to amyloid states can be achieved through a combination of deposition and condensation pathways^33,34^. The dynamic characteristics of the wild-type protein and the variants are detailed in Supplementary Table 1.

Taken together, we designed eight Mef2D variants containing two to three mutations with distinct impact on β-domain dynamics and higher-order assembly. These mutations have not been identified before and their combinations served to probe the role of the β-domain in muscle cell differentiation.

### β-domain rigidity potentiates transcription activity

**Fig. 2:**
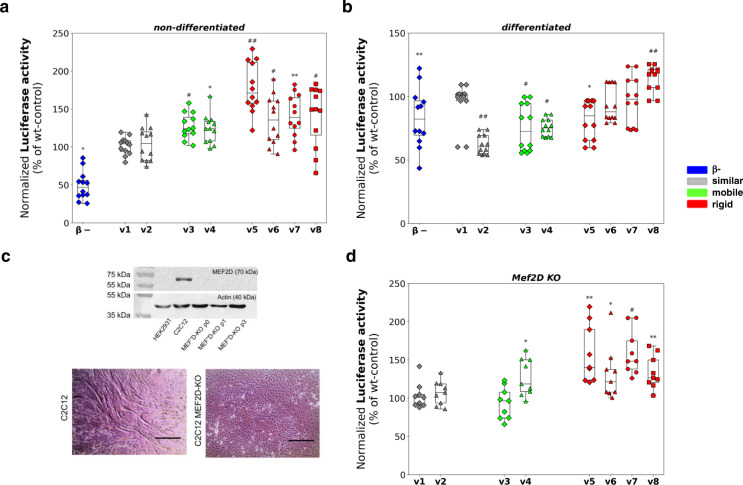
Transcriptional activity of MEF2D variants in C2C12 cells. Luciferase activity normalised to galactosidase signal (Methods) is shown as a percentage of the wild-type (wt) control. Luciferase activity was measured in four biologically independent experiments, using three technical replicates in each with the same samples (*n* = 12 samples in 4 independent experiments). The points represent individual measured data, the rectangles in the box plots present the median and the 25 and 75 percentile values, while the error bars point to 1 and 99%. The luciferase signal is shown as mean  ±  SE, significance (* *p* < 0.05; ** *p* < 0.01; # *p* < 0.005; ## p < 0.001) was computed using two-sided student t-test. The different variants are grouped by their β-domain dynamics properties (Fig. 1c, Methods): **var1** (gray diamond) and **var2** (gray triangle) with similar β-domain dynamics; **var3** (green diamond) and **var4** (green triangle) with mobile β-domain; **var5** (red diamond), **var6** (red triangle), **var7** (red circle) and **var8** (red square) with rigid β-domain as compared to the wild-type. The β- variant is shown by blue diamond. **a** Transcriptional activity in non-differentiated C2C12 myoblasts. Variants with rigid β-domains show significantly higher transcriptional activity then the wild-type (**var5** 178 ± 9.6, **var6** 135.6 ± 9.3, **var7** 141.6±8.1 and **var8** 140.8±11.5 %), while variants with mobile β-domains show slightly increased transcription activity (**var3** 127.7 ± 4.9 and **var4** 123.9 ± 5.5 %) using *n* = 12 samples in 4 independent experiments. Significances (**var3** *p* = 0.0001, **var4** *p* = 0.0012, **var5** *p* = 5.2*10^−6^, **var6** *p* = 0.0027, **var7** *p* = 0.0003, **var8** *p* = 0.0044; β-minus *p* = 1.52*10^−6^) were computed using two-sided student t-test. **b** Transcriptional activity in differentiated C2C12 myotubes**. var8** with rigid β-domain (red square) exhibits significantly higher transcriptional activity than the wild-type, while **var3** (green diamond) and **var4** (green triangle) with mobile β-domains, and **var2** (gray triangle) with similar β-domain dynamics as the wild-type exhibit reduced transcriptional activity. (*n* = 12 samples in 4 independent experiments). Significances (**var2** *p* = 2.9*10^−9^; **var3** *p* = 0.008; **var4** *p* = 1.02*10^−7^; **var8** *p* = 0.0004) were computed using two-sided student t-test. **c** Lack of Mef2D blocks myotube formation in Mef2D knockout C2C12 cell line. Western blot images showing the lack of Mef2D, which are present endogenously in C2C12 cells. Three chosen stable KO cultures were followed through several passages to prove the stable lack of Mef2D (~70 kDa), while actin was used as inner control (~40 kDa). After 6 days of differentiation myotubes form in C2C12 cell line with endogeneous Mef2D (control, left panel), while cannot be observed in MEF2D KO cultures (right panel). Images were taken by transmitted microscopy, scale bars represent 400 µm. **d** Transcriptional activity in C2C12 KO cells. Rigid β-domain variants (red) have higher transcriptional activity than variants with similar dynamics to the wild-type (gray). Significances (**var4** *p* = 0.0175, **var5** *p* = 0.0096, **var6** *p* = 0.0365; **var7** *p* = 0.0022; **var8** *p* = 0.0071) were computed using two-sided student t-test using *n* = 9 samples in 3 independent experiments).

First, we measured the transcriptional activity of Mef2D variants in cycling and differentiated C2C12 cells. The variants were cloned into pCMV eukaryotic expression vectors and were transfected together with two reporter plasmids: MCK-Luc and pSV-βgal (Methods). Transcription efficiency was characterised by the luciferase activity of the variants, which was normalised to the galactosidase signal. In non-differentiating cells, all variants with rigid β-domains (***var5*** – ***var8***) exhibited increased transcriptional activity as compared to the wild-type protein (Fig. 2a). Interestingly, ***var3*** and ***var4*** with mobile β-domains also exhibited slightly elevated transcriptional activity (Fig. 2a). In differentiated C2C12 cells, however, only the ***var8*** exhibited significantly higher transcription activity as compared to the wild-type Mef2D (Fig. 2b). The β- variant was less potent to activate transcription in accord with previous results^13^ (Figs. 2a, 2b).

Then we measured the transcriptional activity without the endogenous Mef2D background using knock-out (KO) cultures generated by CRISPR/Cas9 technique^36^ (Methods). The C2C12 KO cell line was not capable to generate multinucleated myotubes in the absence of Mef2D (Fig. 2c). The transcriptional activity of the variants without the endogenous wild-type background exhibited similar trends as observed in non-differentiated C2C12 cells. The variants with rigid β-domain (***var5*** – ***var8***) potentiated transcription as compared to the wild-type Mef2D, while the variants with similar β-domain dynamics as the wild-type (***var1***, ***var2***) showed comparable activity (Fig. 2D). The variants with mobile β-domain did not exhibit a uniform behavior, while the transcriptional activity of ***var3*** was slightly decreased, that of ***var4*** considerably increased as compared to the wild-type, indicating a more complex mechanism.

Transcriptional activity negatively correlated with the structural disorder of the β-domain (Supplementary Table 2), suggesting that its rigidity promotes interactions with the transcriptional apparatus.

### β-domain rigidity promotes early myotube development

**Fig. 3:**
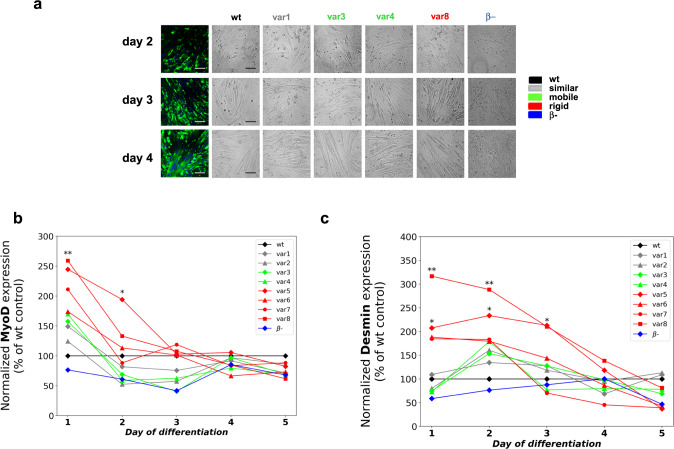
Differentiation progress in the presence of Mef2D variants. **a** Early stage of myotube development (day 2 - day 4). The number of multinucleated, long myotubes in the presence of overexpressed Mef2D variants. Representative fluorescent and transmitted images represent randomly selected visual fields and were used to determine the fusion index of the appropriate cultures. Each experiment was independently repeated two times with similar results, at least 15 randomly selected visual fields were analysed. Scale bar is 50 µm. **b**, **c** Protein expression of myogenic regulatory factors MyoD (**b**) and Desmin (**c**). Normalized protein expression during the differentiation of C2C12 cells. Protein expression was plotted as a percentage of their wild-type control in each day of differentiation. Data was derived from quadruplicate measurements (*n* = 4 independent experiments), significances (*p = 0.0032; **p = 0.0172 for panel **b**, and *p = 0.0149; **p = 0.0002 for panel **c**) were computed using two-sided student t-test as compared to the wild-type control on the given day of differentiation. **b** Protein expression of the early differentiation regulator MyoD. Variants with rigid β-domains (**var5**-**var8**, red) exhibit higher level of MyoD expression on day 1 and day 2. Significant deviations (**var5** *p* = 0.0032; **var8** *p* = 0.0172) were observed in case of **var5** and **var8** using two-sided student t-test as compared to the wild-type control on the given day of differentiation. **c** Protein expression of the late differentiation marker desmin. More rigid β-domain variants (**var5** *p* = 0.0149; **var8** *p* = 0.0002, red) significantly increase desmin expression on days 1 and 2.

In the next step, we assessed the impact of the different variants on skeletal muscle differentiation followed by desmin immunocytochemistry and transmission light microscopy (Methods, Fig. 3). From day 2, we observed multinucleated myotubes with wild-type Mef2D and all variants, and to lesser extent in case of the β- variant. A marked difference between different variants appeared in particular on day 3, when more rigid variants promoted myotube formation as compared to the wild-type (Fig. 3a). At the end of the differentiation process a similar amount of myotubes were formed with all variants, except the one lacking the β-domain (Fig. 3a). The progress of differentiation was quantified by the fusion index, defined as the ratio of the nuclei number in multinucleated myocytes versus the total number of nuclei within the visual fields (Methods, Supplementary Fig. 3). Quantitative analysis corroborated that variants with rigid β-domain, in particular ***var8***, promoted the early phase of differentiation (Supplementary Fig. 3). In addition, ***var4*** with mobile β-domain, also showed a faster myotube development, which was not observed in the presence of ***var3*** (Supplementary Fig. 3).

Mef2D physically interacts with myogenic regulatory factors^8,9^, thus we monitored their protein expression levels by Western blot analysis at each day of the differentiation process. We found increased levels of both the early differentiation regulator MyoD (Fig. 3b) and the late differentiation marker desmin (Fig. 3c) in the presence of rigid β-domain variants at the early phase of differentiation (days 1 and 2). The impact on desmin was prolonged also on day 3 of differentiation (Fig. 3c). This suggests that more ordered β-domains stabilise Mef2D interactions with the myogenic regulatory factors contributing to potentiation of transcription^10^. Along this line, we did not find higher desmin or MyoD levels in the presence of ***var4*** with mobile β-domain (Figs. 3b, 3c), although this variant also exhibited progress in myotube development.

Taken together variants with rigid β-domain promote the early phase of differentiation and stabilise the myogenic factors.

### Mef2D forms higher-order assemblies in C2C12 cells with both liquid-like and solid-like properties

**Fig. 4:**
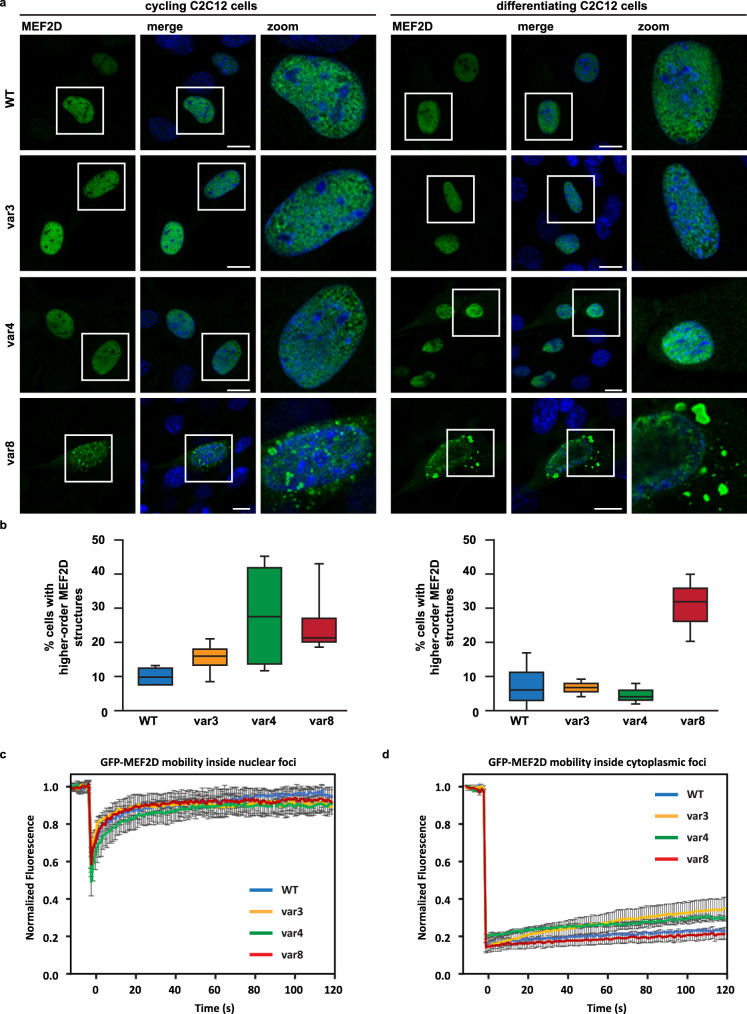
Mef2D forms liquid-like and solid-like higher order assembly. **a** Mef2D foci in the nucleus and cytoplasm. Subcellular distribution of Mef2D **wt**, **var3**, **var4** and **var8** in C2C12 cells grown in cycling or differentiating medium exhibit foci formation in both the nucleus and cytoplasm. Higher-order assembly is most pronounced in case of **var8** with rigid β-domain, but is also observed in case of **var3** and **var4** with mobile β-domain. 24 hrs post-transfection cells were fixed and stained with an antibody specific for Mef2D. Nucleic acid was stained using DAPI. The scale bar is 10 μm on the representative images. The experiment was performed four times (cycling medium) and three times (differentiating medium). Quantification is shown in panel **b**. **b** Quantification of MEF2D cells with cytoplasmic higher-order structures (foci). The percentage of Mef2D overexpressing cells with cytoplasmic aggregates is significantly higher in case of **var8** with rigid β-domain. Cycling C2C12 cells: *n* = 4, ± s.e.m.; differentiating C2C12 cells: *n* = 3 independent experiments, ± s.e.m. Total number of cells counted > 150. Significances were computed by one-way ANOVA followed by Bonferroni-Holm Posthoc in reference to **wt**: **var3** *p* = 0.13 (cycling) and *p* = 0.87 (differentiated), **var4** *p* = 0.09 (cycling) and *p* = 0.60 (differentiated), **var8** *p* = 0.04 (cycling) and *p* = 0.04 (differentiated). **c**, **d** Analysis of mobility of Mef2D higher-order assemblies in nuclear foci (**c**) and cytoplasmic foci (**d**). Mobility was assessed by fluorescence recovery after photobleaching (FRAP) performed after 24 hours post-transfection of GFP-tagged MEF2D wt, **var3**, **var4** and **var8** in C2C12 cells. The mean of the FRAP curve +/- standard error of the mean (s.e.m.) is shown. **c** Number of nuclear foci analyzed: **wt** (3); **var3** (3); **var4** (3); **var8** (3). **d** number of cytoplasmic aggregates analyzed: **wt** (11); **var3** (10); **var4** (10); **var8** (9). All Mef2D proteins show high mobility inside the nuclear foci (**c**) and nucleoplasm (Supplementary Fig. 5b). This is in sharp contrast with the low mobility inside cytoplasmic foci (**d**).

Then we analyzed the cellular organisation of representative Mef2D variants and overexpressed the wild-type, ***var3***, ***var4*** and ***var8*** in C2C12 cells grown under cycling conditions and in presence of differentiation medium. Rapid turnover and accumulation upon inhibition of the proteasome indicates that Mef2D and its variants are disordered^37^ (Supplementary Fig. 4a, Methods). Confocal microscopy revealed that wild-type Mef2D and ***var3***, ***var4*** and ***var8*** formed higher-order assemblies, which are mainly located inside the nucleus (Fig. 4a). Fluorescence recovery after photobleaching (FRAP) analysis indicated high mobility of the wild-type protein as well as ***var3***, ***var4*** and ***var8*** inside the nucleoplasm (Supplementary Fig. 4b) and nuclear foci (Fig. 4c), resembling liquid-liquid phase separated condensates^38^. FRAP analysis in U2OS cells corroborated the presence of mobile nuclear condensates (Supplementary Fig. 5), in case of all variants, irrespective of the β-domain dynamics. These results are in line with the FuzDrop predictions^26^ that Mef2D and all the variants are capable of spontaneously undergoing liquid-liquid phase separation (*p*_*LLPS*_ ~ 0.99).

In addition, we observed cytoplasmic foci reminiscent of higher-order structures^24^ (Fig. 4a). In contrast to the nuclear condensates, the cytoplasmic foci exhibited low mobility (Fig. 4d), indicating solid-like states. The percentage of cells displaying cytoplasmic higher-order assemblies was significantly higher for ***var8*** with rigid β-domain, both in cycling and differentiating myoblasts compared to wild-type Mef2D (Fig. 4b). Surprisingly, we also observed that ***var4*** with mobile β-domain tends to promote the formation of cytoplasmic aggregates in cycling cells (Fig. 4b). The increased propensity of ***var8*** and ***var4*** to form higher-order structures was not due to higher expression levels, since we found similar protein levels in C2C12 cells (Supplementary Fig. 4a).

As both ***var8*** and ***var4*** promoted the early phase of myotube development, we reasoned that ordered higher-order assemblies facilitate myogenesis. In accord, we found a negative correlation between the transcription activity in non-differentiating cells and the probability of β-domain to form liquid-like condensates (Supplementary Table 2).

### Structure and dynamics of Mef2D model peptides

To provide molecular insights how the β-domain modulates the Mef2D higher-order assembly, we have employed different NMR methods and molecular dynamics simulations (Methods) using a 37-residue peptide model of the wild-type Mef2D and representative variants (***var3***, ***var4*** and ***var8***) containing the β-domain and its flanking regions (UniProt code: Q14814, 265-301 residues, Fig. 1b).

Order parameters (S^2^) derived from the backbone chemical shifts based on the Random Coil Index (RCI)^39^ indicated the disordered nature of all peptides (Supplementary Fig. 6a). ***var8*** exhibited an increased ordering in particular at D29 and W31 mutant residues. Higher R2/R1 values of ***var8***, in particular at the C-terminal region suggested conformational exchange between compact and extended conformers corroborating partial ordering (Supplementary Fig. 6b). Diffusion ordered spectroscopy (DOSY) indicated a faster diffusion of ***var8*** (D = 2.95*10^−10^ ± 1.15*10^−10^ m^2^/s) than the wild-type peptide (D = 1.54*10^−10^ ± 2.61*10^−12^ m^2^/s) suggesting more compact conformations (Supplementary Fig. 6c). In contrast, lower diffusion coefficient of ***var3*** (Supplementary Fig. 6c) suggested more extended structures, consistently with the decreased order parameters (Supplementary Fig. 6a).

**Fig. 5:**
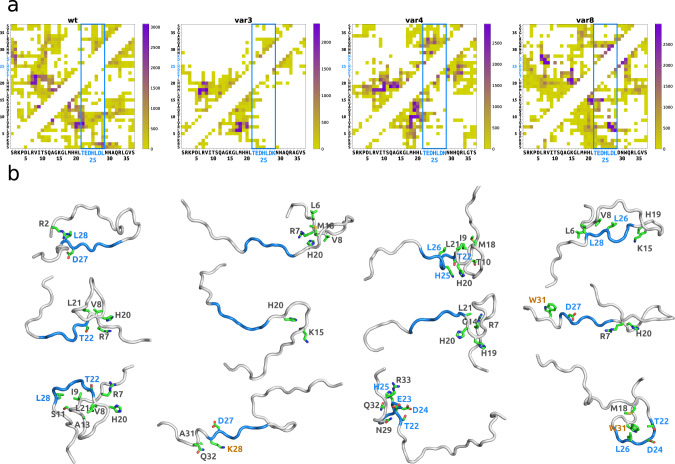
Representative structural features of Mef2D variant peptides from molecular dynamics simulations. Conformational analysis was performed using the 70-100 ns trajectory of each replica (9000 snapshots) (Methods). **a** Contacts maps of the clusters. Mef2D **wt**, **var3**, **var4** and **var8** peptides exhibit distinct intra-molecular interaction patterns (Methods). The β-domain (blue) contributes to structure organisation of **var8** and to lesser extent to **var4**, while does not form persisting contacts in **var3** (see also Supplementary Fig. 7). Color scales indicate the number of snapshots in the clusters, with the given contact sampled. **b** Representative structures of the of Mef2D variant peptides. More compact structures are formed through interactions of the β-domain (blue), such as in case of **var8** and **var4**, while extended structures, such as in case of **var3** sample variable interactions outside the β-domain. β-domain residues are displayed in blue, residues mutated in the different variants are orange labelled.

In accordance with the NMR data, molecular dynamics simulations indicated that the studied Mef2D model peptides (wild-type, ***var3***, ***var4*** and ***var8***) sample a large regime of their conformational space (Supplementary Fig. 7a). In particular, the disordered Mef2D peptides adopt both compact and extended conformations (Supplementary Fig. 7b, Methods) and exhibit distinct contact patterns depending on their β-domain properties (Figs. 1c, 1d, Methods). The characteristic contacts in ***var8*** model peptide are formed dominantly within the β-domain (56 % of all contacts), through a consecutive set of residues (Fig. 5a). The β-domain less frequently contributes to intra-molecular contacts in ***var4*** and the wild-type peptide (34 % and 36% of the contacts, respectively) and is rarely involved in structure formation of ***var3*** (Fig. 5a). In line with the NMR data, structure analysis indicates that ***var8*** forms more compact structures (Fig. 5b, Supplementary Fig. 7b) mediated by hydrophobic contacts (Leu26 and Leu28 residues) and partly aromatic interactions (His25 and Trp31, Fig. 5b, Supplementary Fig. 7c). ***var4*** can sample both compact conformations mediated by polar and aromatic contacts (Thr22, His25 and Asn30 residues), as well as extended conformations mediated by charge-charge interactions mostly with the C-terminal region (Glu23 and Arg33, Fig. 5b, Supplementary Fig. 7b and c). The compact conformations of the wild-type peptide were also mediated by hydrophobic interactions, which are less frequent than those in ***var8*** (Fig. 5b, Supplementary Fig. 7b). In addition, the wild-type peptide also forms charge-charge interactions with the N-terminal region (eg. Asp27-Arg2, Fig. 5b). In contrast, ***var3*** rarely samples compact conformations and forms some local, transient contacts at both the C- and N-terminal regions (Fig. 5b, Supplementary Fig. 7b).

Structure analysis of model peptides suggests that different biophysical forces shape the conformational landscape of the Mef2D variants, and mutations in the β-domain remarkably change the structural variability and compactness of the peptide models.

## Discussion

Elaborating the relationship between the protein state and its function presents a new paradigm in understanding of cellular processes^40^. Transcriptional programmes controlling cell-line identity are frequently regulated by high density of transcriptional apparatus^41,42^, often generating distinct compartments^43^. These transcriptional clusters, also termed as super-enhancers^23^, are stabilised by dynamic and heterogeneous interactions^22^. In general, the material states of such higher-order assemblies must be precisely controlled^24,35^. Solidifying the liquid-like state of a-kinase anchoring protein AKAP95, for example, has been shown to interfere with alternative splicing and initiate tumorigenesis^44^, while ASC interactions are conditioned to the hardening of NLRP6 condensates^45^.

Skeletal muscle development may also involve transcriptional clusters^46^ formed by myogenic proteins and Mef2^2,8^. Proximity of their DNA-binding sites (Supplementary Fig. 8) promotes physical interactions between these factors^8,10^, which is required to activate transcription of muscle-specific genes^46^. We reasoned that the alternative spliced, acidic β-domain contributes to the orchestration of myogenic regulatory factors. Based on sequence analysis and structure predictions (Fig. 1), we anticipated that the β-domain serves as an ordered interaction element within the disordered transactivation region (Supplementary Fig. 1). Thus, by introducing mutations into the β-domain and its flanking region based on FuzDrop predictions, we aimed at modulating Mef2D higher-order assembly.

We have found a negative correlation between the transcriptional activity of the Mef2D variants and β-domain dynamics (Fig. 2, Supplementary Table 2); in particular rigid β-domain variants (***var5*** –***var8***) were more potent to activate transcription in C2C12 cells before differentiation (Fig. 2). Along these lines, the increased transcription activity of the rigid β-domain variants also promoted the early phase of myotube development (Fig. 3, Supplementary Fig. 3), also reflected by altered expression of MyoD and desmin (Fig. 3). Surprisingly, ***var4*** with mobile β-domain also promoted transcription and early phase differentiation (Figs. 2, 3, Supplementary Fig. 3), without increasing the expression of myogenic regulatory proteins (Fig. 3).

We observed that Mef2D forms both nuclear and cytoplasmic foci (Fig. 4). The mobility of the nuclear foci in both C2C12 and U2OS cells resembled mobile nuclear condensates formed through liquid-liquid phase separation (Fig. 4c, Supplementary Figs. 4 and 6). In contrast, we observed the lack of mobility in the cytoplasmic foci (Fig. 4d), indicating the presence of solid-like aggregates. Formation of solid-like assemblies were promoted by ***var8*** with rigid β-domain, as well as by ***var4*** with mobile β-domain (Fig. 4b). ***var4*** was predicted to spontaneously undergo liquid-liquid phase separation as well as to convert between liquid-like and solid-like states (Fig. 1d, Supplementary Table 1). The presence of higher-order structures with both liquid-like and solid-like characters may be explained by two molecular mechanisms. In the first scenario, these assemblies are formed independently, the liquid-like condensates through phase separation and the solid-like aggregates through ordered assembly or deposition^33,34^. In the second scenario, the maturation of the liquid-like condensates leads to the formation of solid-like aggregates. The distinct behaviors of the mobile β-domain variants ***var3*** and ***var4*** seem to be consistent with the second scenario. These two variants exhibit remarkably different cellular context-sensitivity as indicated by the droplet landscape (Fig. 1d). The context-dependent ***var4*** is more potent to activate transcription and promotes differentiation as compared to the less context-dependent ***var3*** (Figs. 2, 3), indicating that a transition of Mef2D between liquid-like and solid-like states may be required during myogenesis. We cannot exclude the possibility that the liquid-like and solid-like states are formed independently due to the different cellular milieu in the nucleus and cytosol. In this case, however, formation of these higher-order assemblies must be regulated through independent pathways during muscle cell differentiation. Instead, we favor the simpler model, in which the transition between the liquid-like and solid like states is required for potentiation of transcriptional programmes during myogenesis.

Based on these results, we propose that transcription potentiation of Mef2D is linked with its higher-order assembly, which enables simultaneous interactions with both the basal machinery and the myogenic factors to activate distinct gene expression programs for muscle lineage development. Our results support the model that Mef2D assemblies are formed by disordered interactions of the transactivation domain. The alternatively spliced β-domain serves as an ordered interaction element to stabilise the disordered assembly. The sensitivity of the β-domain and its flanking regions to the cellular context may facilitate switching between ordered and disordered interactions and, thereby, the adaptation of this segment to the cellular conditions during different stages of the muscle development. Along these lines, we observe both liquid-like and solid-like Mef2D assemblies in C2C12 cells, raising the possibility of their interconversion. The transcription activation and differentiation progress by the droplet-promoting and context-dependent ***var4***, in contrast to the context-insensitive ***var3***, conforms to this scenario. A similar mechanism likely operates during myofibrillogenesis to organise Z-disc formation by FATZ condensates^47^.

In summary, our results shed light on how dynamic properties of an alternatively spliced segment fine-tune Mef2D higher-order assembly to the cellular context, providing a complex regulatory mechanism for gene-expression during skeletal muscle development.

## Methods

### Computational design

We considered the human Mef2D sequence (UniProt code: Q14814; https://legacy.uniprot.org/uniprot/Q14814) and computed three residue-specific dynamics characteristics: *i)* structural disorder in the unbound state using the Espritz method (*p*_*D*_)^30^ as implemented into the FuzPred algorithm (https://fuzpred.bio.unipd.it) *ii)* disordered interactions as predicted by the FuzPred method (*p*_*DD*_)^25^ *iii)* droplet-promoting probability as predicted by the FuzDrop method (*p*_*DP*_)^26^ and we averaged the values of dynamics descriptors (*p*_*D*_*, p*_*DD*_*, p*_*DP*_) for the β-domain (residues 286-292). We introduced mutations in 286-301 residues and computed the differences between the mutant and wild-type β-domain dynamics: *i)* structural disorder Δ*p*_*D*_ = *p*_*D*_(mut) - *p*_*D*_(wt); *ii)* disordered interactions Δ*p*_*DD*_ = *p*_*DD*_(mut) - *p*_*DD*_(wt); *iii)* droplet-promoting probability Δ*p*_*DP*_ = *p*_*DP*_(mut) - *p*_*DP*_(wt), where each probability value is the average of the respective probabilities of the β-domain residues. Mutations with largest impact on the β-domain Δ*p*_*D*_ and with most diverse chemical nature were selected (Fig. 1B). We also considered the droplet-promoting probability (Δ*p*_*DP*_) and the multiplicity of binding modes (MBM) for ranking, to generate variants with distinct higher-order assembly^31^. The droplet landscape was used to inform on the cellular behavior of the higher-order assembly, in particular the likelihood of changing between liquid-like and solid-like higher-order assemblies^33,34^ (Fig. 1D). The variants included two sequences with similar β-domain dynamics to the wild-type (***var1***, ***var2***), two variants with more mobile β-domain (***var3***, ***var4***), and four variants with more rigid β-domain (***var5*** - ***var8***) as compared to the wild-type (Figs. 1B, 1C, 1D). The dynamics characteristics of the variants are summarised in Supplementary Table 1.

### Cell culturing and differentiation

HEK293 (human embryonic kidney) cells and mouse immortalized C2C12 myoblast cell lines (both from ATCC) were cultured in High Glucose DMEM (Biosera, Nuaille, France) supplemented with 10% fetal bovine serum (Gibco by Life Technologies, Carlsbad, California, USA), 1% Penicillin-Streptomycin (Gibco), and 1% L-glutamine (Biosera). The medium was changed every other day, and cells were sub-cultured at 80-90% confluence. The culturing media was exchanged to DMEM containing 2% horse serum (Gibco) to differentiate C2C12 myoblasts into multinucleated myotubes. Differentiation was followed by transmission microscopy for 6 days.

### CRISPR-Cas9 experiments

C2C12 cells were transfected with *MEF2D* specific CRISPR/Cas9 knockout and HDR plasmid constructs, targeting 3 different places in coding sequence (in exon# 3, 4 and 5; Santa Cruz Technology, Dallas, TX, USA). Forty-eight hours after the lipid-mediated transfection puromycin selection (1.5 µg/ml) was applied for 5 days than single cells were sorted by the presence of GFP and PRF expressed from the KO and HDR vectors, respectively, using FACS Aria III flow cytometer (BD Biosciences). *MEF2D*-KO colonies were grown from individual single cells.

### Determination of transcription activity, reporter plasmids

MEF2D variants with modified dynamical properties were cloned into pCMV-HA eukaryotic expression vector (Genscript, Piscataway, NJ, USA). To determine the transcriptional activity of Mef2D variants, MCK-Luc reporter plasmid (Addgene, Watertone, MA, USA) and pSV-β-galactosidase control vector (Promega) were used together with the variant plasmids. Control and MEF2D-KO cells (description for the generation of KO cultures is in the section above) were transfected with the vectors of the variants using Lipofectamine 2000 transfection reagent (Life Technologies, Carlsbad, CA, USA). 48 hours later luciferase and galactosidase activity were determined by Luciferase and β-galactosidase enzyme assays (both from Promega) following the manufacturer´s instructions. Luminescent and absorbance data were collected by Synergy H1 microplate reader to quantify the transcriptional activity of the samples. Microsoft Excel 2016 was used to process transcription and differentiation data.

### Immunocytochemistry and calculation of fusion index

Cultured cells were fixed with 4% PFA followed by washing with 0.1 M glycine in PBS to neutralize excess formaldehyde. Cells were permeabilized with 0.25% Triton-X (Sigma-Aldrich) and blocked with PBS containing 1% BSA. The samples were incubated with primary antibodies (anti-desmin, 1:1000) diluted in blocking solution overnight at 4 °C. Following the application of fluorophore-conjugated secondary antibodies (Biotinylated anti-mouse IgG (1:300), Alexa Fluor 488 conjugated Streptavidin (1:100); both from Thermo Fisher Scientific) mounting medium containing DAPI (Vector Laboratories, Burlingame, USA) was added to each coverslip. Immunolabelled samples were investigated with a Zeiss LSM510 laser scanning confocal microscope (Zeiss, Oberkochen, Germany). Fusion index was quantified as the ratio of the nuclei number in myocytes with two or more nuclei versus the total number of nuclei within a visual field.

### Immunofluorescence on cultured cells

C2C12 cells were grown on polylysine-coated glass coverslip. C2C12 cells were transfected using Lipofectamine 3000 (Life Technologies) following manufacturer instructions. 24 hrs post-transfection, cells were washed with cold PBS and fixed with 3.7% formaldehyde in PBS for 9 minutes at room temperature, followed by permeabilization with ice-cold acetone for 5 minutes at −20 °C. PBS containing 3% BSA and 0.1% Triton X-100 was used for blocking and incubation with primary (MEF2D, 14353-1-AP, Proteintech) and secondary antibodies (Alexa Fluor® 488, A‐21202, Thermo Scientific). Images were obtained using a Leica TCS-SP8 (Leica Microsystems) and a 63x oil immersion lens.

### Western blot analysis

For Western blot analysis cells were harvested in lysis buffer, the protein content of samples was measured by a BCA protein assay (Pierce, Rockford, IL, USA). Protein samples were separated in 7.5% SDS–polyacrylamide gels, then transferred to nitrocellulose membranes (Whatman, Maidstone, England). After blocking non-specific binding sites, membranes were exposed to primary antibodies overnight at 4 °C as follows: mouse monoclonal anti-MEF2D (1:500; BD Biosciences, San Jose, CA, USA), monoclonal mouse myoD (Novus Biologicals, Abington, UK) antibody in 1:200 dilution, mouse monoclonal anti-desmin in 1:1000 dilution (BD Biosciences) and goat polyclonal anti-actin (1:250; Santa Cruz Biotechnology, Dallas, USA). Following incubation with horseradish peroxidase-conjugated, respective anti-goat or anti-mouse IgG antibodies (Bio-Rad, Hercules, CA, USA; 1:1000, 1 hour) the immunoreactive bands were visualized by enhanced chemiluminescence (Pierce or Millipore, Billerica, MA, USA). The optical density of signals was measured using a Kodak Gel Logic 1500 system and further analyzed by using ImageJ 1.40g freeware.

### Confocal microscopy and Fluorescence recovery after photobleaching (FRAP) analysis

FRAP measurements on C2C12 and U2OS cells transfected with GFP-tagged Mef2D WT, var3, var4 and var8 were performed using a confocal microscope Leica TCS SP8 (Leica Systems). For FRAP analysis we used a 63x oil immersion objective. Bleaching was obtained by using a laser intensity of 100% at 405 nm for 1 s. Recovery was recorded for 120 time points after bleaching (120 s). Analysis of the recovery curves were carried out with the FIJI/ImageJ. Fluorescent density analysis was performed using FIJI/ImageJ and selecting specific region of interest (ROI). The flow of the protein was measured by quantifying the recovery of the bleached area at the cost of the unbleached region and using a custom written FIJI/ImageJ routine. The bleached region was corrected for general bleaching during image acquisition. We quantified the molecules that move from the unbleached region to the bleached region, leading to recovery of the bleached region. Prior to FRAP analysis, the images were corrected for drift using the StackReg plug‐in function of the FIJI software suite. The equation used for FRAP analysis is: ((Ibleach − Ibackground)/(Ibleach(t0) − Ibackground(to)))/((Itotal‐Ibackground)/(Itotal(t0) − Ibackground(to))), where Itotal is the fluorescence intensity of the entire cellular structure, Ibleach represents the fluorescence intensity in the bleach area, and Ibackground the background of the camera offset. FRAP curves were averaged to obtain the mean and standard error mean.

### Graphics and statistics

Pooled data were expressed as mean ± standard error of the mean (SEM). The mean and SEM were calculated as weighted averages and weighted standard errors with weight corresponding to the number of samples and number of experiments. Box plots and line graphs were constructed using Origin 8.6 graphic software. In all box-plot type presentations the rectangles in the box plots present the median and the 25 and 75 percentile values, while the error bars point to 1 and 99%. The differences between control cultures and cells overexpressing Mef2D variants were assessed using one-way analysis of variance (ANOVA) and student’s t-test function of Microsoft Excel 2016, the significance and a *p* value of less than 0.05 was considered statistically significant. Vector graphics were prepared by Inkscape 0.92.5 (2060ec1f9f, 2020-04-08), https://www.inkscape.org.

### Molecular Dynamics (MD) simulations

#### Model system

The starting models of the 37 residue peptides of the wild-type protein, ***var4*** and ***var8*** terminated by acetyl (ACE) and N-methyl (NME) groups were generated by the LEaP module of AMBERTools17^48^. The solute was neutralised by adding Na^+^ and Cl^-^ ions randomly and then solvated by 12 Å of TIP3 water^49^. Periodic boundary conditions using a truncated octahedron geometry with cell edge of 151 Å was used with particle-mesh Ewald method^50^ for long-range electrostatic interactions and 8 Å non-bonded interaction cut-off. All simulations were performed using a Charmm36m force field, which was developed for intrinsically disordered proteins^51^.

#### Computational protocol

Extended conformations of the models were subjected to minimization by steepest descent and conjugate gradient methods. The system was heated from 0 K to 50 K in 50 ps using Langevin thermostat, then from 50 K to 310 K in 1 ns using Berendsen thermostat while applying a harmonic restraint of 4 kcal mol^−1^/Å^2^ for all atoms. Water density was adjusted to ~1 g/cm^3^ in 500 ps NPT simulation using Berendsen barostat, applying 1 kcal mol^−1^/Å^2^ restraint on the backbone atoms, which was then released for 1 ns NPT simulation. Then three independent 100 ns NPT simulations at 310 K and 1 bar were carried out for each model, with 2 fs timestep applying the SHAKE algorithm on the hydrogen geometry. Coordinates were saved in each 10 ps. The translational center of mass motions was removed in a wrapping procedure every 1000 steps.

#### MD analysis

The MD trajectories were analysed using the MDTraj Python package v1.97^52^. We considered the 70 to 100 ns trajectory of each replica based on the RMSD values in reference to the average structure (Supplementary Fig. 7). Radius of gyration of was computed based on the method developed for disordered proteins^52^, using MDTraj Python package. Clustering of the conformations of the disordered peptides was based on the contact map similarity^31^. Contacts were computed using GetContacts (https://getcontacts.github.io, commit: b0777f7148a327387133d9632f8ed1e34bfaa282). We defined the similarity between two contact maps as: ${f}_{S}=\frac{{C}_{i}\cap {C}_{j}}{{(C_{i} \cup {C}_{j})}-(C_{i}\cap {C}_{j})}$, where C_i_ and C_j_ are the set of contacts in snapshot *i* and *j*, the numerator is the common contacts of the two sets and the denominator is the total number of different contacts of the two sets. We clustered two snapshots if *f*_*S*_ > 0.5, and the clusters were joined based on the frequencies of the common contacts. For graphical clarity, we visualised contacts only in the four most populated clusters. The calculations were performed with Python (https://pypi.org) and Numpy (https://numpy.org) libraries, plots were prepared using the matplotlib python package (https://matplotlib.org/). Structure visualisation was done using PyMol (The PyMOL Molecular Graphics System, Version 2.0 Schrödinger, LLC).

### NMR analysis

37-residue peptide sequences of the wild-type, ***var3***, ***var4*** and ***var8*** containing the β-domain and its flanking residues (UniProt code: Q14814, 265-301 residues) were ^15^N labelled on leucines (as underlined SRKPDLRVITSQAGKGLMHHLTEDHLDLNNAQRLGVS). All NMR experiments were carried out on a Bruker Avance Neo (700 MHz ^1^H frequency) spectrometer equipped with a Prodigy TCI cryoprobe, and the temperature was set to 298 K. Chemical shifts were referenced to DSS. Peptides were dissolved in 5% D_2_O and 95% H_2_O 20 mM PBS buffer solution, with a final peptide concentration of 1 mM. For NMR data acquisition and evaluation, Bruker’s Topspin 4.0 software package was utilized.

#### NMR resonance assignment

2D correlation spectra, including ^1^H-^1^H TOCSY and ^1^H-^1^H ROESY with ^15^N decoupling, ^13^C-^1^H HSQC, and ^13^C-HSQC-TOCSY, ^15^N-^1^H HSQC, ^15^N-^1^H HSQC-TOCSY, and -ROESY.

#### Determination of order parameters (S^2^) from chemical shifts

Chemical shifts (C_α_, C_β_, and H_α_) were used to extract Random Coil Indices and NMR order parameters (S^2^). First, the offset-corrected data were converted to secondary shifts, then site-specific corrections were applied to calculate Random Coil Indices^53^. Residue order parameters (S^2^) were derived from the chemical shifts of Cα, Cβ, and Hα backbone atoms^39^.

#### Relaxation measurements

^15^N-relaxation data (T_1_, T_2_) were obtained by Bruker pseudo 3D ^15^N-^1^H correlation experiments with double INEPT-transfer and decoupling during acquisition. The relaxation delay times were set to 10, 20, 40, 80, 120, 160, 200, 300, 400, 600, 1200, and 2400 ms for T_1_ and the Carl-Purcell-Meiboom-Gill (CPMG) pulse trains of 17, 34, 51, 68, 85, 102, 119, 153, 204, 254, and 305 ms were used for T_2_-measurements. 64 increments were collected, and the number of transients was 8 for T_1_/T_2_-measurements and 80 for the heteronuclear (^15^N-^1^H) NOE experiment to obtain a proper S/N ratio. Reduced Spectral Densities^54^ and R2/R1 ratios of ^15^N nuclei of isotopically labeled leucine residues were derived from relaxation data (^15^N T_1_, T_2_ and NOE).

#### Diffusion ordered spectroscopy

*(DOSY)* Diffusion coefficients were derived from a measurement with a stimulated spin echo LED (Longitudinal-Eddy-current Delay) sequence with bipolar gradients and two spoil gradients^55^. The length of the diffusion delay (75 ms) and gradient pulses (2 ms) were optimized by short, one-dimensional (1D) experiments. The gradient strength was split to 32 increments, linearly varying between 5 % and 95 % of its maximum value. The maximum gradient strength of the probe was 57.7 G/cm. The number of scans was set to 16. Data processing was performed and 2D DOSY plots were generated by the dosy module of Topspin. Diffusion coefficients were calculated by an exponential fit of the signal intensity decays obtained with increasing gradient strength.

### ChIP-Seq Protocol

C2C12 myoblasts and myotubes from late differentiation stage were cross-linked using disuccinimidyl glutarate (DSG, Sigma) followed by fixation with 1% methanol-free ultrapure formaldehyde (Thermo Scientific). After washing, cells were harvested in ChIP lysis buffer and centrifuged at 12,000g for 1 min at 4 °C. Resuspended chromatin samples were sonicated to get fragments between 100 and 2000bp. Chromatin IP was performed using anti-MEF2D antibody (NBP-1-80567, Novus Biologicals) with overnight incubation at 4 °C. Antibody-chromatin complex was centrifuged (3,500 rpm, 20 min, 4 °C), then samples were incubated with paramagnetic beads (Dynabeads Protein A, Thermo Scientific) for 6h at 4 °C. Antibody-chromatin-bead complexes were washed with different buffers, then DNA-protein complexes were eluted and de-cross-linked by adding NaCl (0.2 M). ChIPed DNA was purified using Qiagen MinElute columns and prepared for sequencing according to the manufacturer’s instructions (Illumina).

### ChIP-seq data analysis

The library was sequenced at the Genomic Medicine and Bioinformatics Core Facility at the University of Debrecen, Hungary using single end 75 bp Illumina technology. The raw reads were aligned to the mm10 reference genome sequence using Burrows-Wheeler Aligner. The resulted file was further analyzed using the Homer approach^56^, involving the peak calling, the denovo motif finding and the annotation of the peaks and motifs under the peaks. The top 1000 peaks based on the Homer’s peak scores were analyzed for denovo motifs. The most enriched motifs, the hlf motif (https://jaspar.genereg.net/matrix/MA0043.2/), and the Mef2A motif were used to annotate the peaks in the Mef2D enhancer region. In addition to Mef2D, transcription factors binding to the creatine kinase gene (Mck) enhancer region were derived from the ChIPSummitDB database (https://summit.med.unideb.hu/summitdb/).

### Reporting summary

Further information on research design is available in the Nature Portfolio Reporting Summary linked to this article.

## Supplementary information

Supplementary information

Reporting Summary

## Data Availability

Source data are provided with this paper. The data that support the findings are available in the data repository of University of Debrecen at 10.48428/ADATTAR/3RFQRC. ChIP-seq sequencing data can be accessed in the SRA database of NCBI project number PRJNA887931, and GEO accession code GSE224053. Transcription factors binding to the creatine kinase gene (Mck) enhancer region used for ChIP-seq data analysis were derived from the ChIPSummitDB database (https://summit.med.unideb.hu/summitdb/). Mef2D sequence was derived from the UniProt database (https://www.uniprot.org/) Q14814 (main isoform). NMR assignments have been deposited in BMRB database with the codes BMR51840 [10.13018/BMR51840] (wt), BMR51841 [10.13018/BMR51841] (var3), BMR51842 [10.13018/BMR51842] (var4), BMR51843 [10.13018/BMR51843] (var8). Molecular dynamics trajectories were deposited as 10.5281/zenodo.7657119. Source data are provided with this paper.

## Source data

Source data

## Code availability

The scripts used to process the trajectories and the results generated by MDTraj Software version 1.9.7 and GetContacts (https://getcontacts.github.io/commit:b0777f7) as well as to produce the plots used in the article are available: https://drive.google.com/drive/folders/1IDFZwTUmoCPy4T_B_AGRKCL2OQq_rZOC. The link also contains the processed MD trajectories used for analysis. The underlying libraries are: Python, Numpy (https://numpy.org) and matplotlib (https://matplotlib.org/).
