# Supplementary material for: Alternatively spliced exon regulates context-dependent MEF2D higher-order assembly during myogenesis
Source: Nat Commun. 2023 Mar 10;14:1329. doi: 10.1038/s41467-023-37017-7 (PMC10006080; doi:10.1038/s41467-023-37017-7)
Supplement: Supplementary file 1 — Supplementary information [file 41467_2023_37017_MOESM1_ESM.pdf]

Alternatively spliced exon regulates context-dependent MEF2D higher-order assembly during myogenesis

Mónika Gönczi[1,2], João M.C. Teixeira[3], Susana Barrera-Vilarmau[3], Laura Mediani[4], Francesco Antoniani[4], Tamás Milán Nagy[5,6], Krisztina Fehér[5,6], Zsolt Ráduly[1,2], Viktor Ambrus[7], József Tőzsér[7], Endre Barta[7], Katalin E. Kövér[5,6], László Csernoch[1,2], Serena Carra[4*], Monika Fuxreiter[3]*

[1]*Department of Physiology, Faculty of Medicine, University of Debrecen, Egyetem tér 1, H-4032, Debrecen, Hungary*
[2]*ELKH Cell Physiology Research Group, Department of Physiology, Faculty of Medicine, University of Debrecen, Egyetem tér 1, H-4032 Debrecen, Hungary*

[3]*Department of Biomedical Sciences, University of Padova,Via Ugo Bassi 58/B, 35131 Padova, Italy*

[4]*Department of Biomedical, Metabolic and Neural Sciences, University of Modena and Reggio Emilia G. Campi 287, 41125, Modena, Italy*

[5]*Department of Inorganic and Analytical Chemistry, University of Debrecen, Egyetem tér 1, H-4032 Debrecen, Hungary*

[6]*MTA-DE Molecular Recognition and Interaction Research Group, University of Debrecen Egyetem tér 1, H-4032 Debrecen, Hungary*
[7]*Department of Biochemistry and Molecular Biology, Faculty of Medicine, University of Debrecen, Egyetem tér 1, H-4032 Debrecen, Hungary*

*Correspondence: serena.carra@unimore.it, monika.fuxreiter@unipd.it

Supplementary tables

Supplementary Table 1. Computed dynamic parameters of the Mef2D variants. Structural disorder (p_D) was computed using the Espritz method[1]; disordered binding (p_{DD}) by the FuzPred method[2]; droplet-promoting probability (p_{DP}) by the FuzDrop method[3]; multiplicity of binding modes (MBM) by the FuzPred method[4]. The values are averaged for three regions, β-domain containing 286-292 residues, β-domain flanking region containing 270-301 residues, β-domain extended flanking region containing 250-301 residues.

Supplementary Table 2. Correlation between the dynamics and transcriptional activity. The Spearman correlation coefficients between the normalised Luciferase activities in non-differentiated (**Figure 1b, Table S1**) and differentiated (**Figure 1c, Supplementary Table S1**) cells and dynamics in the unbound state (p_D, computed by the FuzPred program [2]) as well as the probability of forming a liquid-like higher-order state (p_{DP}, computed by the FuzDrop method[3]). The predicted dynamics parameters were averaged for the β-domain.

Supplementary Table 3. Primer pairs used for qPCR analysis. Genes and protein names, and sequences (5' $\rightarrow$ 3') are displayed.

Supplementary Table 1. Computed dynamic parameters of the Mef2D variants. Structural disorder (p_D) was computed using the Espritz method[1]; disordered binding (p_{DD}) by the FuzPred method[2]; droplet-promoting probability (p_{DP}) by the FuzDrop method[3]; multiplicity of binding modes (MBM) by the FuzPred method[4]. The values are averaged for three regions, β-domain containing 286-292 residues, β-domain flanking region containing 270-301 residues, β-domain extended flanking region containing 250-301 residues.

**variant**	**Structural disorder (p_D)**	**Disordered binding (p_{DD})**	**Droplet probability(p_{DP})**	**Multiplicity of BM (*MBM*)**
**β-domain 286-292 residues**				
bmin	0.4336	0.4552	0.6476	0.835
wt	0.1796	0.2424	0.3093	0.629
var1	0.1823	0.5621	0.4832	0.760
var2	0.3273	0.1717	0.4121	0.600
var3	0.4799	0.0597	0.5046	0.189
var4	0.5033	0.1621	0.5855	0.517
var5	0.1423	0.4318	0.3711	0.745
var6	0.1592	0.4125	0.3769	0.725
var7	0.1282	0.2288	0.2605	0.649
var8	0.1303	0.3448	0.3154	0.682
**β-domain and flanking 270-301 residues**				
bmin	0.4297	0.5866	0.6666	0.712
wt	0.2636	0.3537	0.4469	0.697
var1	0.2938	0.5185	0.5450	0.781
var2	0.3700	0.2168	0.4786	0.614
var3	0.5212	0.2201	0.6146	0.514
var4	0.5043	0.3221	0.6358	0.680
var5	0.3417	0.4207	0.5421	0.736
var6	0.2787	0.4372	0.4950	0.741
var7	0.1819	0.3144	0.3547	0.670
var8	0.2344	0.3704	0.4256	0.720
**β-domain and flanking 250-301 residues**				
bmin	0.5201	0.6236	0.7460	0.708
wt	0.4111	0.4948	0.6117	0.700
var1	0.4401	0.5974	0.6767	0.750
var2	0.4862	0.3810	0.6308	0.658
var3	0.5904	0.3491	0.7133	0.610
var4	0.5738	0.4300	0.7268	0.689
var5	0.4667	0.4797	0.6630	0.746
var6	0.4301	0.5328	0.6426	0.726
var7	0.3610	0.4606	0.5532	0.681
var8	0.3938	0.5065	0.5986	0.709

Supplementary Table 2. Correlation between the dynamics and transcriptional activity. The Spearman correlation coefficients between the normalised Luciferase activities in non-differentiated (**Figure 1b**) and differentiated (**Figure 1c**) cells and dynamics in the unbound state (p_D, computed by the FuzPred program[2]) as well as the probability of forming a liquid-like higher-order state (p_{DP}, computed by the FuzDrop method[3]). The predicted dynamics parameters were averaged for the β-domain (**Supplementary Table S1**). Higher-order assembly plays a more important role in regulating gene-expression programs at the early stage of differentiation. Significances were determined by two-sided Spearman's rank correlation test.

**Parameter**	**Non-differentiated**		**Differentiated**	
	R	***p***	***R***	***p***
p_D	-0.65	0.07	**-0.73**	0.03
p_D (without var8)	-0.57	0.15	**-0.67**	0.08
p_{DP}	**-0.78**	0.02	-0.57	0.12
p_{DP} (without var8)	**-0.76**	0.03	-0.43	0.30

Supplementary Table 3. Primer pairs used for qPCR analysis. All primers were purchased from Sigma Aldrich, Saint Louis, MI, USA.

Gene name	***Protein name***	***Sequence (5´-3´)***
**Myogenin**	Myogenin	AGTGAATGCAACTCCCACAGC<br>TATCCTCCACCGTGATGCTGT
**APP**	amyloid beta (A4) precursor protein	CCGAGAGAGAATGTCCCAGGT<br>AAGCTGCTGTCTCTCATTGGC
**Prkaca**	protein kinase, cAMP dependent, catalytic, alpha	GGAACTGGGCTTGGAATCTCG<br>GTAATTTCCCCAGCAGCTCCC
**Cyclophyllin**	Cyclophillin	TGGAGAGCACCAAGACAGACA<br>TGCCGGAGTCGACAATGAT
**18S**	18S Ribosomal RNA	TCGAGGCCCTGTAATTGGAAT<br>TCCCAAGATCCAACTACGAGCTT

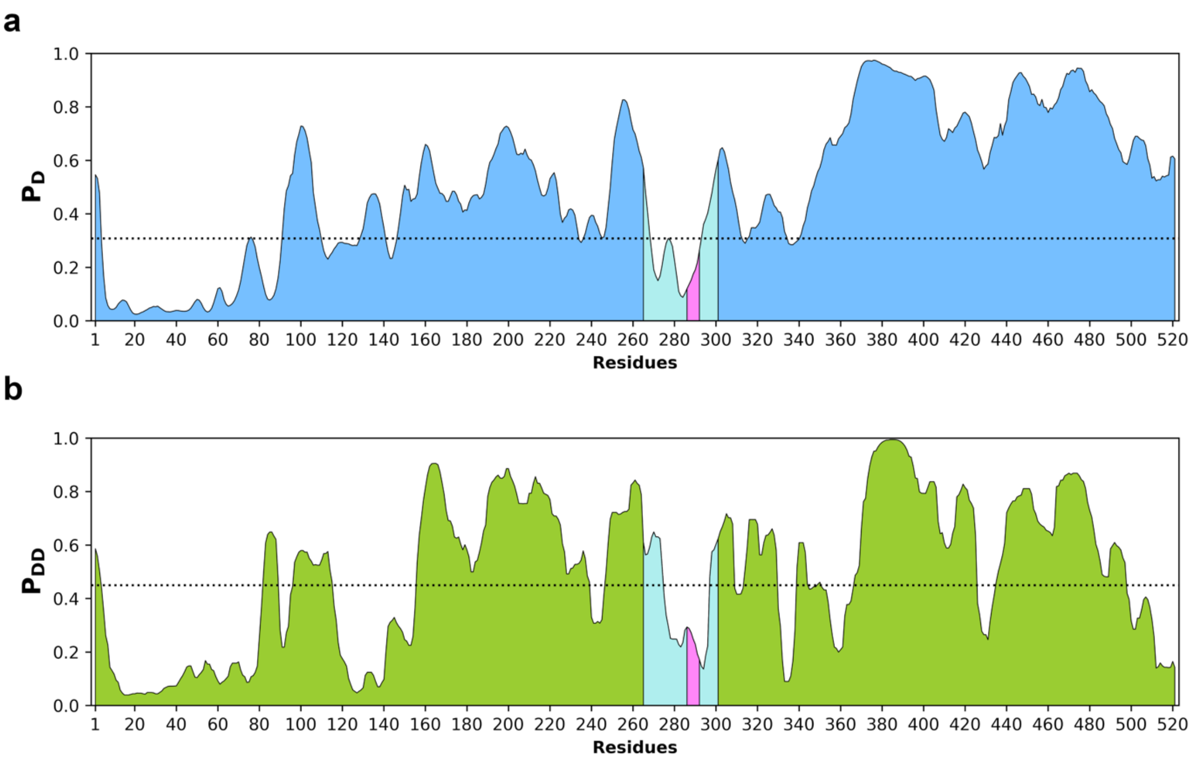

Supplementary Figure 1. Predicted dynamics characteristics of Mef2D (UniProt code: Q14814) in free and bound states. (a) Predicted structural disorder. *Residue-specific disorder scores by the Espritz method[1] indicating that the β-domain (magenta) and its flanking regions (cyan) have increased structural stability within the disordered transactivation region (from residue 87). The horizontal line at $p_D \geq 0.3085$ indicates the threshold between order/disorder[1].* ***(b) Probability of disordered interactions.*** *Based on the FuzPred method[2], the majority of transactivation region have high probability of forming disordered, heterogeneous interactions (p_{DD})[5]. In addition to the β-domain, the 239-247, 331-339, 355-363, 427-434 residue regions serve as stable interaction elements. These can serve as regulatory motifs, K245 was identified as an acetylation site[6].*

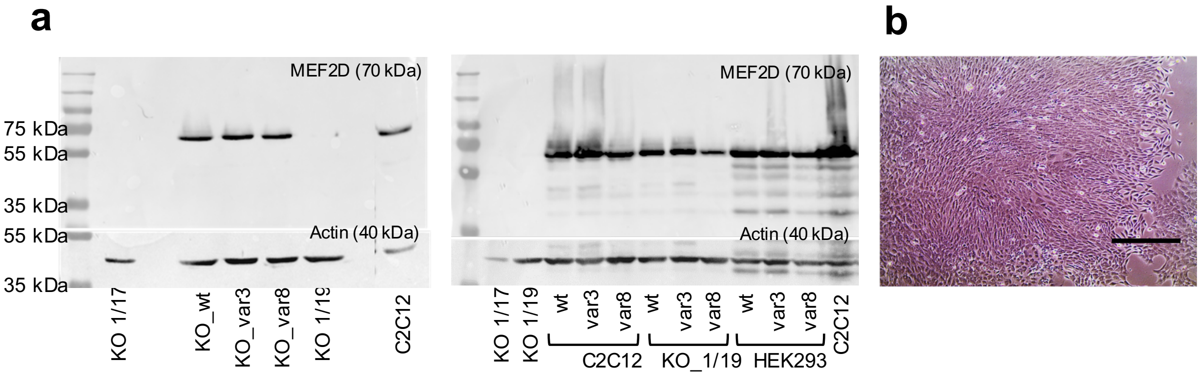

Supplementary Figure 2. Transient overexpression of MEF2D variants and its effect on MEF2D-KO C2C12 cultures. (a) *Representative Western blot images regarding the successful transient transfection of MEF2D variants into KO cultures using pCMV vector construct. Each experiment was repeated independently three times with similar results (as shown in* ***Figure 2d****). Non-transfected knockout cells and control C2C12 samples were used as a negative and positive control, respectively, to probe the specificity of the MEF2D antibody. The molecular weight of MEF2D protein is 70 kDa, while actin-specific antibody was used as a normalizing control (40 kDa). Different MEF2D KO clones were used for the transfections.* ***(b)*** *MEF2D-KO culture, transfected with pCMV-Mef2D* ***var8****, and differentiated for 5 days. Each transfection experiment was repeated three times with similar results, in each case one parallel culture was subjected into serum deprivation-induced differentiation. Scale bar represents 400 µm.*

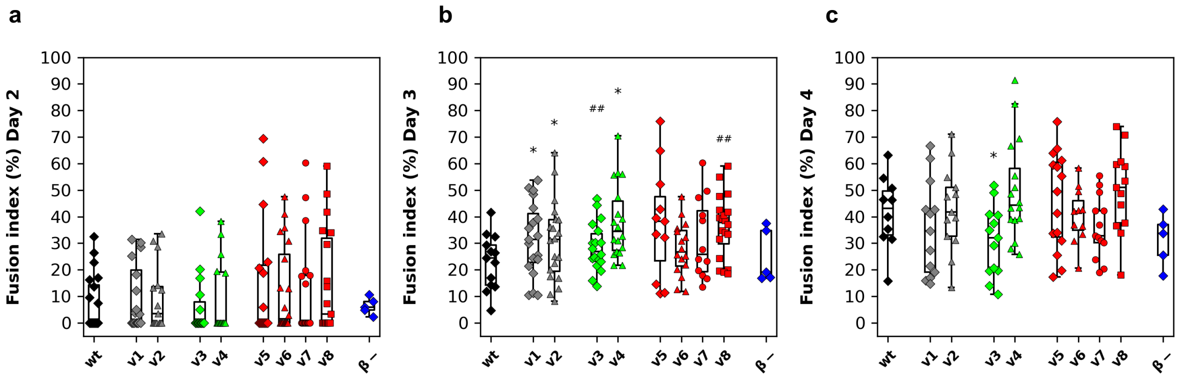

Supplementary Figure 3. Myotube development on day 2 (a) day 3 (b) and day 4 (c) in the presence of different Mef2D variants. *Pooled data of the fusion index, defined as the ratio of the nuclei number in multinucleated myocytes versus the total number of nuclei within the visual fields determined from day 2 to day 4 following the transfection of individual MEF2D variants. The points represent individual measured values, the rectangles in the box plots present the median and the 25 and 75 percentile values, while the error bars point to 1 and 99%. The different variants are grouped by their β-domain dynamics properties (**Figure 1c, Methods**): **var1** (gray diamond) and **var2** (gray triangle) with similar β-domain dynamics to the wild-type; **var3** (green diamond) and **var4** (green triangle) with mobile β-domain; **var5** (red diamond), **var6** (red triangle), **var7** (red circle) and **var8** (red square) with rigid β-domain as compared to the wild-type. The significance (*p<0.05; **p<0.01; ##p<0.001) was computed using student t-test. Significant differences are observed on day 3 based on n=2 biologically independent experiments with at least 5 randomly selected visual fields were performed in each experiment. Significance on day3 was (**var1** p=0.027; **var2** p=0.044; **var4** p=0.0007; **var5** p=0.0250; **var8** p=0.0009) was computed using two-sided student t-test, while on day 4 (**var3** p=0.03).*

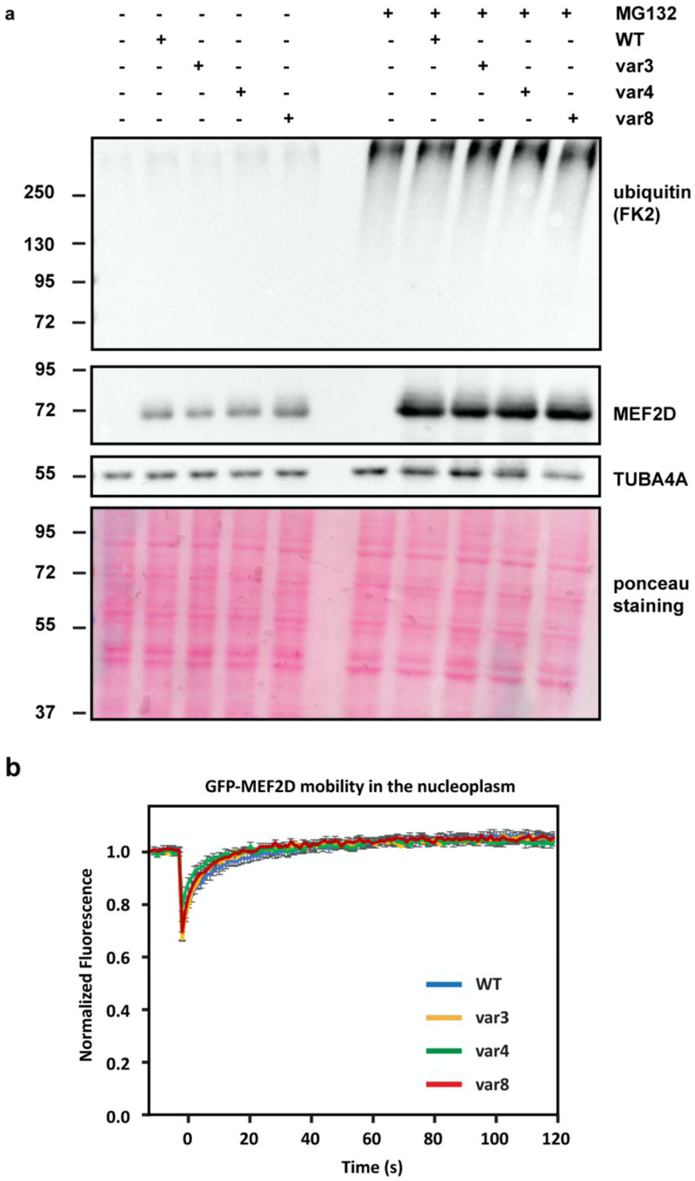

Supplementary Figure 4. Dynamics of Mef2D in native and higher-order state. (a) Mef2D is disordered and susceptible to degradation. *Protein extracts were prepared from cycling C2C12 cells overexpressing either an empty vector (-), MEF2D* ***wt****,* ***var3****,* ***var4*** *or* ***var8****. Where indicated cells were treated with MG132 (20 µM) for 8 hrs prior to cell lysis. Immunoblotting showing the expression levels of MEF2D and polyubiquitinated proteins (FK2 antibody). TUBA4A and ponceau staining are shown as loading controls.* ***(b) Mef2D forms highly mobile higher-order assemblies in nucleoplasm.*** *Mobility was assessed by fluorescence recovery after photobleaching (FRAP) performed after 24 hours post-transfection of GFP-tagged MEF2D wt,* ***var3****,* ***var4*** *and* ***var8*** *in C2C12 cells. The mean of the FRAP curve +/- standard error of the mean (s.e.m.) is shown. Number of ROI analyzed in the nucleoplasm: wt (11);* ***var3*** *(10);* ***var4*** *(10****); var8*** *(11). All MEF2D proteins show high mobility inside the nucleoplasm.*

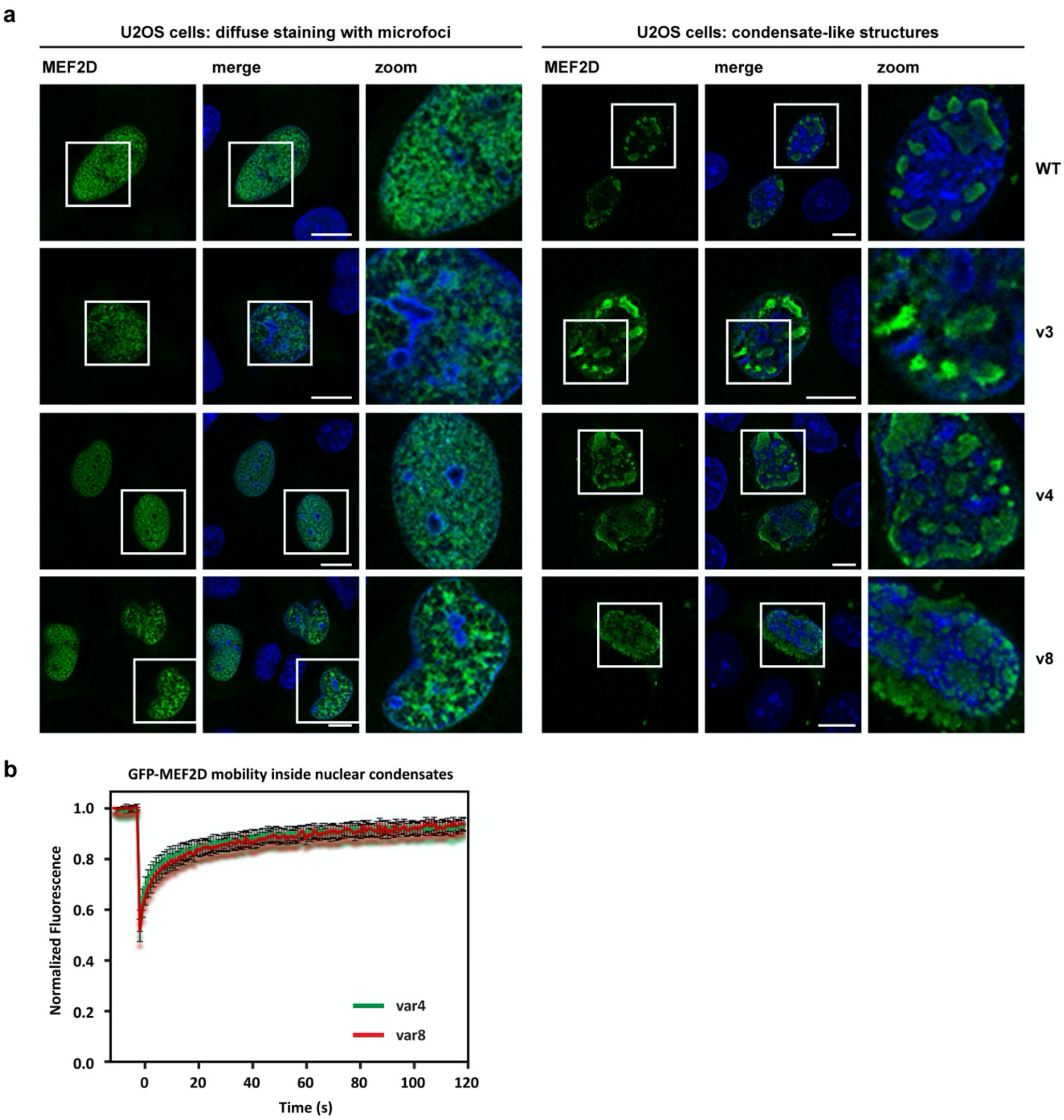

Supplementary Figure 5. Dynamics of Mef2D higher-order assemblies in U2OS cells. (a) Nuclear foci of Mef2D resemble biomolecular condensates in U2OS cells. *Confocal microscopy images of the subcellular distribution of MEF2D wt, var3, var4 and var8 in U2OS cells. In the nucleus, MEF2D WT and its variants form puncta, which resemble those of biomolecular condensates formed through liquid-liquid phase separation* [7, 8]*. Representative images. The experiment was performed three times.* ***(b) High mobility of nuclear Mef2D foci in U2OS indicates the presence of liquid-liquid phase separated condensates.*** *Fluorescence recovery after photobleaching (FRAP) analysis shows that higher-order structures of both var4 and var8 in the nucleus are highly mobile, indicating their liquid-like character. The mean of the FRAP curve +/- standard error of the mean (s.e.m.) is shown. Number of nuclear condensates analyzed: var4 (12); var8 (8).*

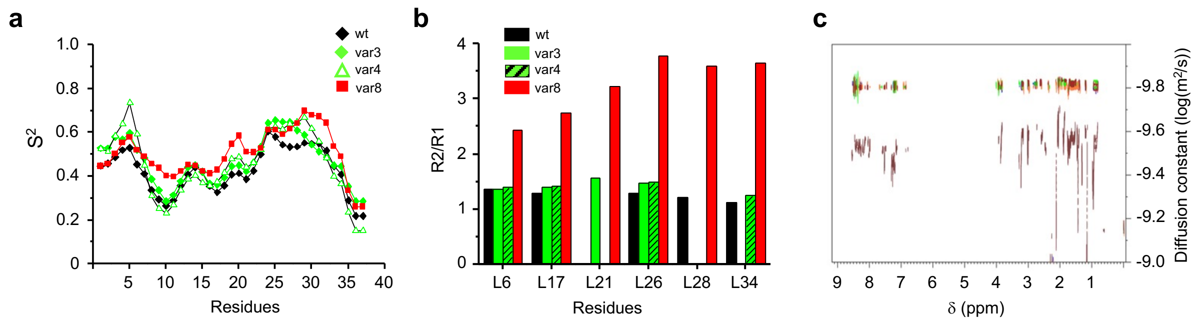

Supplementary Figure 6. Characterisation of the Mef2D β-domain peptides by NMR. *Dynamics of a 37-residue peptide sequence containing the β-domain and its flanking residues (UniProt code: Q14814, 265-301 residues), N^{15} labelled on leucines in the wild-type, **var3**, **var4** and **var8** sequences were studied by different NMR methods. **(a) Order parameters.** Backbone dynamics was characterized by residue order parameters (S^2), which were derived from the chemical shifts of Cα, Cβ, and Hα backbone atoms[9]. **var8** (red square), in particular regions 18-22 residues, and 29-34 residues exhibit decreased dynamics as compared to the wild-type (black diamond), **var3** (green diamond) and **var4** (green triangle). **(b) Relaxation rates.** The higher R2/R1 values indicate conformational exchange between compact and extended conformers in particular in the C terminal region of **var8** (red) as compared to **var3** (green), and **var4** (green hatched), as well as the wild-type Mef2D (black). **(c) Diffusion constants.** The higher diffusion constant of **var8** (brown, $D=2.95*10^{-10} \pm 1.15*10^{-10}$ m^2/s) as compared to the **wt** (blue, $D=1.54*10^{-10} \pm 2.61*10^{-12}$ m^2/s), **var3** (orange, $D=1.56*10^{-10} \pm 3.47*10^{-13}$ m^2/s), **var4** (green, $D=1.51*10^{-10} \pm 1.75*10^{-12}$ m^2/s) indicates a more compact structure.*

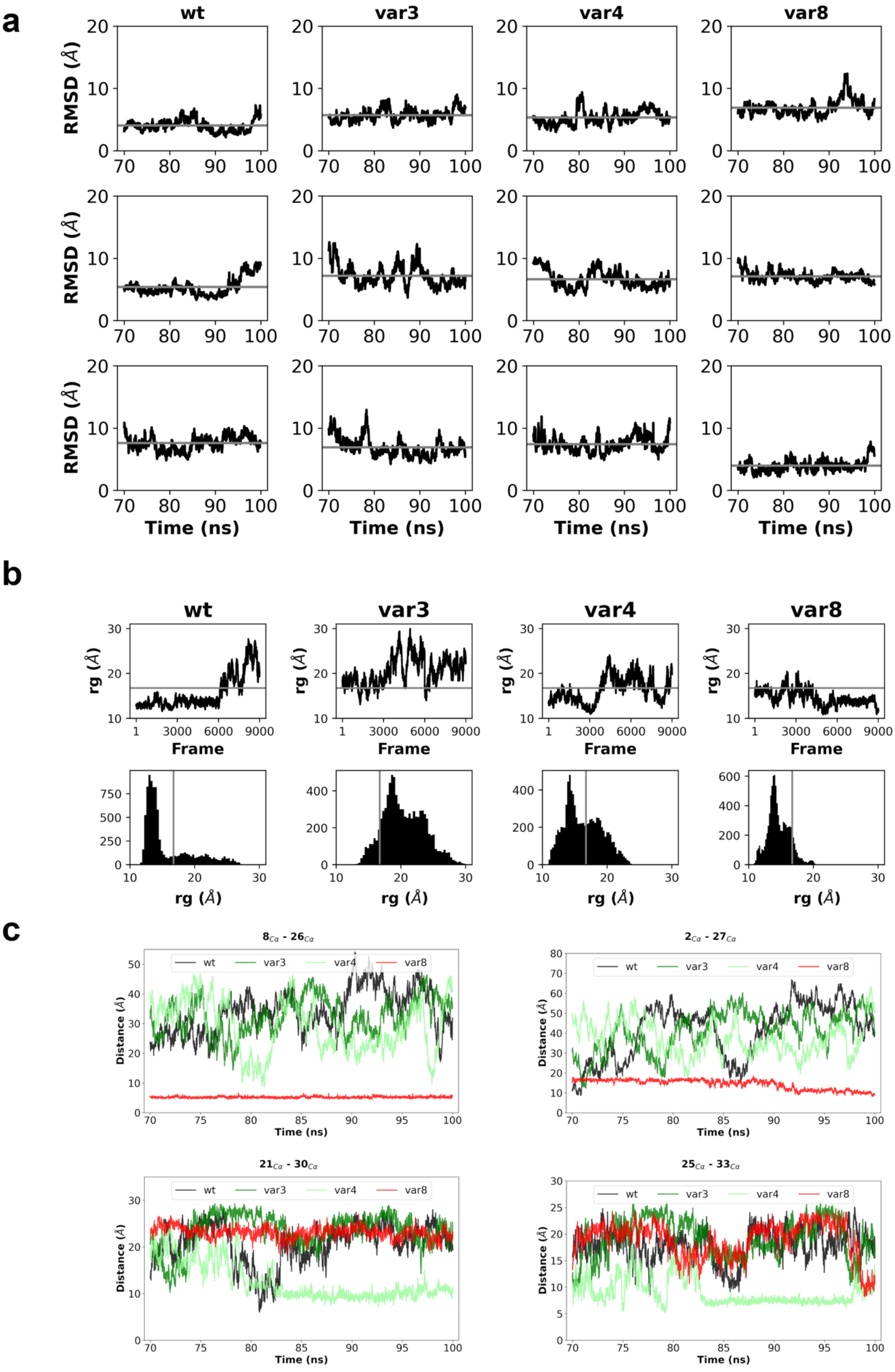
a
wt
var3
var4
var8
RMSD (Å)
Time (ns)
b
wt
var3
var4
var8
rg (Å)
Frame
rg (Å)
c
8Cα - 26Cα
2Cα - 27Cα
21Cα - 30Cα
25Cα - 33Cα
wt
var3
var4
var8
Distance (Å)
Time (ns)

Supplementary Figure 7. Analysis of molecular dynamics trajectories. *Three parallel 100 ns molecular dynamics simulations using 37-residue peptide sequence of the wild-type, **var3**, **var4** and **var8** containing the β-domain and its flanking residues (UniProt code: Q14814, 265-301 residues) were performed (**Methods**), and the last 30 ns of each simulation (70-100 ns) were analyzed.* ***(a) Root-mean-square deviations (RMSD)*** *were computed using the average structure (70-100 ns) as a reference. The mean RMSD values: wt:5.65 $\pm$ 1.46 Å, **var3**: 6.60 $\pm$ 0.64 Å, **var4**: 6.46 $\pm$ 0.86 Å, **var8**: 5.99 $\pm$ 1.45) indicate that all peptides are disordered.* ***(b) Compactness of structure.*** *The radius of gyration was computed using the MDTraj Python library. The theoretical Rg was considered 16.728 Å[14]. Rg indicate compact and extended conformations, the populations of which depend on β-domain dynamics. The Rg histograms (lower panels) were used to inform on the populations of compact (c) and extended (e) structures The vertical line represents a theoretical estimate for compact structures[10] The percentage of compact versus extended conformations: wild type: 71% (c) 29% (e); **var3:** 11% (c) 89% (e); **var4**: 57% (c) 43% (e); **var8**: 89% (c) 11% (e).* ***(c) Time evolution of representative distances.*** *Distances characterising the β-domain interactions (underlined) are shown between the Cα atoms of Val8-<u>Leu26</u> top (left), Arg2-Asp27 (top right), <u>Leu21</u>-Asn30 (bottom left), <u>His25</u>-Arg33 (bottom right) of the wild-type (black), **var3** (dark green), **var4** (light green) and **var8** (red). Hydrophobic interactions are most stable in **var8** (left), while polar and aromatic-charge interactions contribute to structure formation in **var4**.*

a

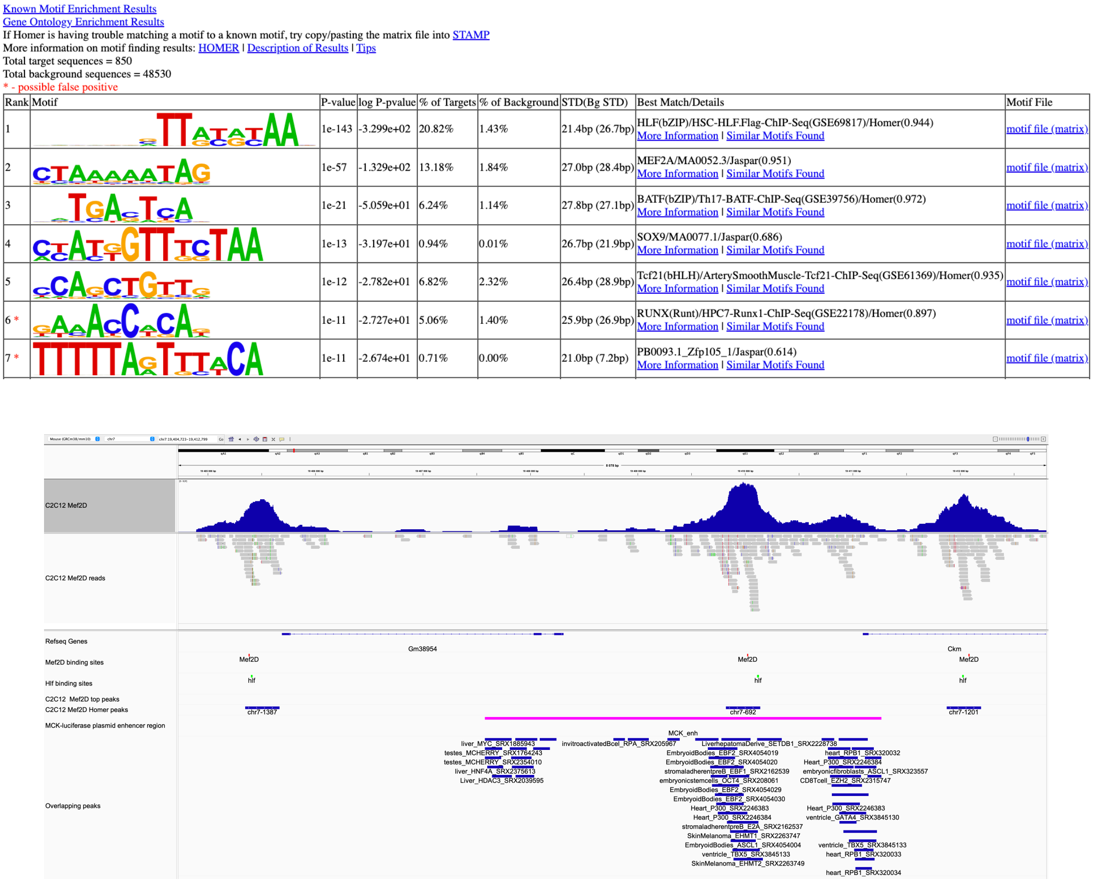

Known Motif Enrichment Results
Gene Ontology Enrichment Results
If Homer is having trouble matching a motif to a known motif, try copy/pasting the matrix file into STAMP
More information on motif finding results: HOMER | Description of Results | Tips
Total target sequences = 850
Total background sequences = 48530
* - possible false positive

Rank	Motif	P-value	log P-pvalue	% of Targets	% of Background	STD(Bg STD)	Best Match/Details	Motif File
1		1e-143	-3.299e+02	20.82%	1.43%	21.4bp (26.7bp)	HLF(bZIP)/HSC-HLF.Flag-ChIP-Seq(GSE69817)/Homer(0.944) More Information | Similar Motifs Found	motif file (matrix)
2		1e-57	-1.329e+02	13.18%	1.84%	27.0bp (28.4bp)	MEF2A/MA0052.3/Jaspar(0.951) More Information | Similar Motifs Found	motif file (matrix)
3		1e-21	-5.059e+01	6.24%	1.14%	27.8bp (27.1bp)	BATF(bZIP)/Th17-BATF-ChIP-Seq(GSE39756)/Homer(0.972) More Information | Similar Motifs Found	motif file (matrix)
4		1e-13	-3.197e+01	0.94%	0.01%	26.7bp (21.9bp)	SOX9/MA0077.1/Jaspar(0.686) More Information | Similar Motifs Found	motif file (matrix)
5		1e-12	-2.782e+01	6.82%	2.32%	26.4bp (28.9bp)	Tcf21(bHLH)/ArterySmoothMuscle-Tcf21-ChIP-Seq(GSE61369)/Homer(0.935) More Information | Similar Motifs Found	motif file (matrix)
6 *		1e-11	-2.727e+01	5.06%	1.40%	25.9bp (26.9bp)	RUNX(Runt)/HPC7-Runx1-ChIP-Seq(GSE22178)/Homer(0.897) More Information | Similar Motifs Found	motif file (matrix)
7 *		1e-11	-2.674e+01	0.71%	0.00%	21.0bp (7.2bp)	PB0093.1_Zfp105_1/Jaspar(0.614) More Information | Similar Motifs Found	motif file (matrix)

b

Supplementary Figure 8. Chip-seq analysis of Mef2D in differentiated C2C12 cells. (a) Analysis of Mef2D DNA binding motifs. *The peak calling analysis of ChIP-seq experiment gave 85990 peaks, out of which 4882 were in the promoter regions of the genes. Most of the peaks were located in the intronic and intergenic regions. The top 1000 peaks were selected based on the Homer peak scores[11]. Based on denovo motif identification, the two most enriched motifs are the HLF motif (https://jaspar.genereg.net/matrix/MA0043.2/) and the Mef2A motif.* ***(b) Analysis of the overlap with other transcription factors****. In differentiated C2C12 cells, Mef2D binding motifs within the Mck enhancer region overlap with other transcription factor binding sites, including MAX, STAT3, JUND, GATA2.*

References

1. Walsh I, Martin AJ, Di Domenico T, Tosatto SC. ESpritz: accurate and fast prediction of protein disorder. *Bioinformatics* **28**, 503-509 (2012).

2. Miskei M, Horvath A, Vendruscolo M, Fuxreiter M. Sequence-Based Prediction of Fuzzy Protein Interactions. *J Mol Biol* **432**, 2289-2303 (2020).

3. Hardenberg M, Horvath A, Ambrus V, Fuxreiter M, Vendruscolo M. Widespread occurrence of the droplet state of proteins in the human proteome. *Proc Natl Acad Sci U S A* **117**, 33254-33262 (2020).

4. Horvath A, Miskei M, Ambrus V, Vendruscolo M, Fuxreiter M. Sequence-based prediction of protein binding mode landscapes *PLoS Comp Biol* **16**, e1007864 (2020).

5. Barrera-Vilarmau S, Teixeira JMC, Fuxreiter M. Protein interactions: anything new? *Essays Biochem* **66**, 821-830 (2022).

6. Choudhary C*, et al.* Lysine acetylation targets protein complexes and co-regulates major cellular functions. *Science* **325**, 834-840 (2009).

7. Boija A*, et al.* Transcription Factors Activate Genes through the Phase-Separation Capacity of Their Activation Domains. *Cell* **175**, 1842-1855 e1816 (2018).

8. Zhu L, Brangwynne CP. Nuclear bodies: the emerging biophysics of nucleoplasmic phases. *Curr Opin Cell Biol* **34**, 23-30 (2015).

9. Cilia E, Pancsa R, Tompa P, Lenaerts T, Vranken WF. From protein sequence to dynamics and disorder with DynaMine. *Nature communications* **4**, 2741 (2013).

10. Bernado P, Blackledge M. A self-consistent description of the conformational behavior of chemically denatured proteins from NMR and small angle scattering. *Biophys J* **97**, 2839-2845 (2009).

11. Heinz S*, et al.* Simple combinations of lineage-determining transcription factors prime cis-regulatory elements required for macrophage and B cell identities. *Mol Cell* **38**, 576-589 (2010).
