# Supplementary material for: Alternatively spliced exon regulates context-dependent MEF2D higher-order assembly during myogenesis
Source: Nat Commun. 2023 Mar 10;14:1329. doi: 10.1038/s41467-023-37017-7 (PMC10006080; doi:10.1038/s41467-023-37017-7)
Supplement: Supplementary file 2 — Reporting Summary [file 41467_2023_37017_MOESM2_ESM.pdf]

nature portfolio

Corresponding author(s): MONIKA FUXREITER
SERENA CARRA

Last updated by author(s): Feb 24, 2023

Reporting Summary

Nature Portfolio wishes to improve the reproducibility of the work that we publish. This form provides structure for consistency and transparency in reporting. For further information on Nature Portfolio policies, see our Editorial Policies and the Editorial Policy Checklist.

Statistics

For all statistical analyses, confirm that the following items are present in the figure legend, table legend, main text, or Methods section.

n/a	Confirmed	
☐	☒	The exact sample size (*n*) for each experimental group/condition, given as a discrete number and unit of measurement
☐	☒	A statement on whether measurements were taken from distinct samples or whether the same sample was measured repeatedly
☐	☒	The statistical test(s) used AND whether they are one- or two-sided<br>*Only common tests should be described solely by name; describe more complex techniques in the Methods section.*
☐	☒	A description of all covariates tested
☒	☐	A description of any assumptions or corrections, such as tests of normality and adjustment for multiple comparisons
☐	☒	A full description of the statistical parameters including central tendency (e.g. means) or other basic estimates (e.g. regression coefficient) AND variation (e.g. standard deviation) or associated estimates of uncertainty (e.g. confidence intervals)
☐	☒	For null hypothesis testing, the test statistic (e.g. *F*, *t*, *r*) with confidence intervals, effect sizes, degrees of freedom and *P* value noted<br>*Give P values as exact values whenever suitable.*
☒	☐	For Bayesian analysis, information on the choice of priors and Markov chain Monte Carlo settings
☒	☐	For hierarchical and complex designs, identification of the appropriate level for tests and full reporting of outcomes
☒	☐	Estimates of effect sizes (e.g. Cohen's *d*, Pearson's *r*), indicating how they were calculated

Our web collection on statistics for biologists contains articles on many of the points above.

Software and code

Policy information about availability of computer code

Data collection	Zeiss LSM510 laser scanning confocal microscope; SYNERGY H1 microplate reader; FACS Aria III flow cytometer; Kodak Gel Logic 1500; Roche LightCycler 480; Bruker TopSpin 4.0 (NMR); Amber 16.0 (Molecular Dynamics simulation); Leica TCS SP8 microscope (Leica Microsystems) equipped with a White Light Laser and with a ×63 oil-immersion lens, Uniprot database (https://www.uniprot.org/) (sequence); ChIPSummitDB database (https://summit.med.unideb.hu/summitdb/) (ChIP-seq analysis);
Data analysis	https://imagej.nih.gov/ij/ (ImageJ 1.40g freeware); Microsoft excel 2016; Origin 8.6; Primer Premier 5.0 software; Primer Blast (http://www.ncbi.nlm.nih.gov/tools/primer-blast); Bruker TopSpin 4.0 (NMR); Dynamics Center 2.7.2 (NMR); Matlab 2 (NMR); Cpptraj 16.16, VMD 1.9.3, Python 3.0 (MD). GitHub repository for MD analysis and visualization: https://github.com/ocel0t/MEF2D; Daniel's XL Toolbox (https://www.xltoolbox.net/), MDTraj Software version 1.9.7; GetContacts (https://getcontacts.github.io/ commit: b0777f7); (The PyMOL Molecular Graphics System, Version 2.0 Schrödinger, LLC); matplotlib python package (Matplotlib - PyPI; https://pypi.org), Numpy python package (NumPy; https://numpy.org) and Python package (https://pypi.org)

For manuscripts utilizing custom algorithms or software that are central to the research but not yet described in published literature, software must be made available to editors and reviewers. We strongly encourage code deposition in a community repository (e.g. GitHub). See the Nature Portfolio guidelines for submitting code & software for further information.

Data

Policy information about availability of data

All manuscripts must include a data availability statement. This statement should provide the following information, where applicable:

- Accession codes, unique identifiers, or web links for publicly available datasets
- A description of any restrictions on data availability
- For clinical datasets or third party data, please ensure that the statement adheres to our policy

Data availability
Source data are provided with this paper. The data that support the findings are available in the data repository of University of Debrecen at https://doi.org/10.48428/ADATTAR/3RFQRC. ChIP-seq sequencing data can be accessed in the SRA database of NCBI project number PRJNA887931 (ncbi.nlm.nih.gov/sra/?term=%20PRJNA887931), and GEO accession code (GSE224053). Transcription factors binding to the creatine kinase gene (Mck) enhancer region used for ChIP-seq data analysis were derived from the ChIPSummitDB database (https://summit.med.unideb.hu/summitdb/). Mef2D sequence was derived from the UniProt database (https://www.uniprot.org/) Q14814 (main isoform). Q14814 (main isoform). NMR assignments have been deposited in BMRB database with the codes BMR51840 (wt), BMR51841 (var3), BMR51842 (var4), BMR51843 (var8). Molecular dynamics trajectories were deposited as https://doi.org/10.5281/zenodo.7657119.
https://doi.org/10.13018/BMR51840 (wt)
https://doi.org/10.13018/BMR51841 (var3)
https://doi.org/10.13018/BMR51842 (var4)
https://doi.org/10.13018/BMR51843 (var8)
Code availability
The scripts used to process the trajectories and the results generated by MDTraj Software version 1.9.7 and GetContacts (https://getcontacts.github.io/ commit: b0777f7) as well as to produce the plots used in the article are available:
https://drive.google.com/drive/folders/1IDFZwTUmoCPy4T_B_AGRKCL2OQq_rZOC
The link also contains the MD trajectories used for analysis. The underlying libraries are: Python, Numpy (https://numpy.org) and matplotlib (https://pypi.org).

Human research participants

Policy information about studies involving human research participants and Sex and Gender in Research.

Reporting on sex and gender	not applicable
Population characteristics	not applicable
Recruitment	not applicable
Ethics oversight	not applicable

Note that full information on the approval of the study protocol must also be provided in the manuscript.

Field-specific reporting

Please select the one below that is the best fit for your research. If you are not sure, read the appropriate sections before making your selection.

- [x] Life sciences
- [ ] Behavioural & social sciences
- [ ] Ecological, evolutionary & environmental sciences

For a reference copy of the document with all sections, see nature.com/documents/nr-reporting-summary-flat.pdf

Life sciences study design

All studies must disclose on these points even when the disclosure is negative.

Sample size	Luciferase activity was measured in four biologically independent experiments, using three technical replicates in each with the same samples (n=12 samples in 4 independent experiments) (Figure 2). Representative fluorescent and transmitted images represent randomly selected visual fields and were used to determine the fusion index of the appropriate cultures. Each experiment was independently repeated two times with similar results, at least 15 randomly selected visual fields were analysed. (Figure 3). Number of nuclear foci analyzed: wt (3); var3 (3); var4 (3); var8 (3). (d) number of cytoplasmic aggregates analyzed: wt (11); var3 (10); var4 (10); var8 (9). (Figure 4). Conformational analysis was performed using the 70-100 ns trajectory of each replica and the three independent trajectories were merged (9000 snapshots), based on the analysis of RMSD from averaged structure (Figure 5). Transient overexpression of MEF2D variants and its effect on MEF2D-KO C2C12 cultures. Each experiment was repeated independently three times with similar results (Figure S2). Myotube development on day 3 based on n=2 biologically independent experiments with at least 5 randomly selected visual fields were performed in each experiment (Figure S3). FRAP experiments: number of nuclear foci wt (3); var3 (3); var4 (3); var8 (3) (Figure 4c) number of cytoplasmic aggregates wt (11); var3 (10); var4 (10); var8 (9) (Figure 4d) in the nucleoplasm: wt (11); var3 (10); var4 (10); var8 (11) (Figure S4) . Dynamics of Mef2D higher-order assemblies in U2OS cells number of nuclear condensates analyzed: var4 (12); var8 (8) (Figure S5).

Data exclusions	All data were included for transcription activity measurements (Figure 2), myotube development (Figure 3, S3), FRAP analysis (Figure 4, S4, S5) and NMR measurements (Figure S6). In MD trajectories the 0-70 ns part of each replica was excluded from the conformational analysis (Figure 5, S7), as these have not reached equilibrium.
Replication	Luciferase activity was measured in four biologically independent experiments, using three technical replicates in each (Figure 2). Fusion index determination experiments were independently repeated two times (Figure3, S3). FRAP experiments: number of nuclear foci wt (3); var3 (3); var4 (3); var8 (3) (Figure 4c) number of cytoplasmic aggregates wt (11); var3 (10); var4 (10); var8 (9) (Figure 4d) in the nucleoplasm: wt (11); var3 (10); var4 (10); var8 (11) (Figure S4) . FRAP in U2OS cells var4 (12); var8 (8) (Figure S5). The DOSY measurements (Figure S6) were repeated two times. The number of transients (replication numbers / number of scans) used for specific experiments providing sufficient signal-to-noise ratios for reliable measurement of signal intensities are given in NMR section of Methods. Molecular dynamics was carried out using three independent replicates.
Randomization	This study was based on computational design, which classified the samples as shown in all analysis by different colors based on the dynamical properties of the beta domain. Apart from this classification, all experimental data , samples and conditions for each variant were varied randomly.
Blinding	We used two kinds of blind reference, the b- variant lacking the b domain (Figure 2, Figure 3, Figure S3). We have also used Mef2D KO cell lines lacking the endogeneous protein, so we could study the variants without the endogeneous background (Figure S2, Figure 2d). Blinding does not apply to NMR since no manual counting or scoring was used to obtain the data.

Reporting for specific materials, systems and methods

We require information from authors about some types of materials, experimental systems and methods used in many studies. Here, indicate whether each material, system or method listed is relevant to your study. If you are not sure if a list item applies to your research, read the appropriate section before selecting a response.

Materials & experimental systems

n/a	Involved in the study
☐	☒ Antibodies
☐	☒ Eukaryotic cell lines
☒	☐ Palaeontology and archaeology
☒	☐ Animals and other organisms
☒	☐ Clinical data
☒	☐ Dual use research of concern

Methods

n/a	Involved in the study
☐	☒ ChIP-seq
☐	☒ Flow cytometry
☒	☐ MRI-based neuroimaging

Antibodies

Antibodies used	mouse monoclonal anti-MEF2D clone 9/MEF2D (BD Biosciences, #610775; Lot:6182887 and 3025570); rabbit polyclonal anti-MEF2D antibody (Novus Biologicals, #NBP-1-80567); mouse monoclonal anti-MyoD (Novus Biologicals, #NB-100-56511 (5.8A)); mouse monoclonal anti-desmin (BD Biosciences, #550626, clone RD301); goat polyclonal anti-actin (Santa Cruz Biotechnology, #sc-1616 (I-19), lot:K1714); HRP-conjugated anti-goat and anti-mouse IgG (Bio-Rad, #1721034 and #1706516); biotinylated rat anti-mouse IgG (Thermo Scientific, #13-4013-85); Alexa Fluor 488 conjugated streptavidin (Thermo Scientific, #S32354, lot: 1719656); MEF2D rabbit polyclonal antibody (Proteintech 14353-1-AP; Highly cross-absorbed secondary antibody Alexa Fluor® 488 (Thermo Scientific, A-21202)
Validation	Specificity of each primary antibodies during the immunofluorescent staining (mouse monoclonal anti-desmin; MEF2D rabbit polyclonal antibody) was tested in negative control staining, where no primary antibody, but only secondary antibody was used. In the Western blot experiments specificity of the antibodies (mouse monoclonal anti-MyoD; mouse monoclonal anti-desmin; goat polyclonal anti-actin) was tested by using protein samples from non-transfected HEK293 cell (which does not express MEF2D and other myogenic factors), and using protein samples from non-transfected C2C12 cells (which express myogenic factors and MEF2D endogenously).

Eukaryotic cell lines

Policy information about cell lines and Sex and Gender in Research

Cell line source(s)	HEK293 (ATCC #CRL-1573), C2C12 cell lines (ATCC #CRL-1772), U-2 OS - HTB-96 | ATCC
Authentication	The original C2C12 cell line authentication procedure included the differentiation protocol, where exchange of culturing media induced the generation of multinucleated myotubes. MEF2D KO C2C12 cell lines were authenticated for the lack of MEF2D continously, at different passage numbers (Figure S2). For HEK293 cells no specific authentication protocol was used, however, the lack of endogenous MEF2D expression in the original cell line, and appropriate overexpression of different MEF2D variants were checked and proven. U2OS was authenticated as described Kedersha N, et al. J Cell Biol. 2020. PMID: 31851327.
Mycoplasma contamination	Here we confirm that the original C2C12 and HEK293 cell line, and also the MEF2D knockout cell line tested negative for mycoplasma contamination.

Commonly misidentified lines (See ICLAC register) | N.A.

ChIP-seq

Data deposition

- [x] Confirm that both raw and final processed data have been deposited in a public database such as GEO.
- [x] Confirm that you have deposited or provided access to graph files (e.g. BED files) for the called peaks.

Data access links<br>*May remain private before publication.*	https://doi.org/10.48428/ADATTAR/3RFQRC<br>ncbi.nlm.nih.gov/sra/?term=%20PRJNA887931<br>NCBI tracking system #23684239, GEO accession number: GSE224053
Files in database submission	SRA database under the PRJNA887931 project number; GSE224053<br>nm_muscle_C2C12_7d_Mef2d_IVDI-homerpeaks-annotations.txt.gz and nm_muscle_C2C12_7d_Mef2d_IVDI-homerpeaks.bed.gz at the repository of the University of Debrecen (https://doi.org/10.48428/ADATTAR/3RFQRC)
Genome browser session<br>(e.g. UCSC)	*Provide a link to an anonymized genome browser session for "Initial submission" and "Revised version" documents only, to enable peer review. Write "no longer applicable" for "Final submission" documents.*

Methodology

Replicates	1
Sequencing depth	n/a
Antibodies	anti-MEF2D antibody (Novus Biologicals, NBP-1-80567)
Peak calling parameters	default
Data quality	n/a
Software	Homer

Flow Cytometry

Plots

Confirm that:

- [ ] The axis labels state the marker and fluorochrome used (e.g. CD4-FITC).
- [ ] The axis scales are clearly visible. Include numbers along axes only for bottom left plot of group (a 'group' is an analysis of identical markers).
- [ ] All plots are contour plots with outliers or pseudocolor plots.
- [ ] A numerical value for number of cells or percentage (with statistics) is provided.

Methodology

Sample preparation	C2C12 cells were transfected with MEF2D-specific CRISPR/KO and HDR plasmids. Fortyeight hours after the transfection procedure puromycin selection (1.5 μg/ml) was applied for 5 days. Selected cultures were trypsinized, collected in normal culture media and single cells expressing both GFP and RFP fluorescence proteins (encoded in the KO and HDR vectors, respectively) were sorted individually into each well of 96 well culturing plates at 37 C.
Instrument	FACS Aria III flow cytometer (BD Biosciences)
Software	Flow cytometry was only used for preparation of the KO cell line, as described above. As the method was not used for data-analysis, no software was applied and no plots related to the method are shown in the paper. The sorting was based on the fluorescent markers, as described below.
Cell population abundance	Double (GFP and RFP) positive cells were further used to generate MEF2D-KO C2C12 cell clones. Individual cell colonies were tested for the lack of MEF2D protein expression.
Gating strategy	For the detection of GFP excitation wavelenght of 488 nm was used, signals were detected at 509 nm. For RFP detection 532 nm excitation and 588 nm emission wavelenghts were used. A figure showing the gating strategy is available at the link: https://doi.org/10.48428/ADATTAR/3RFQRC. It was not included in the Supplement as no analysis was based on the FACS analysis, it was only used for sample preparation.

- [ ] Tick this box to confirm that a figure exemplifying the gating strategy is provided in the Supplementary Information.
